# *Operando* insights into correlating CO coverage and Cu–Au alloying with the selectivity of Au NP-decorated Cu_2_O nanocubes during the electrocatalytic CO_2_ reduction†

**DOI:** 10.1039/d3ey00162h

**Published:** 2023-10-25

**Authors:** Clara Rettenmaier, Antonia Herzog, Daniele Casari, Martina Rüscher, Hyo Sang Jeon, David Kordus, Mauricio Lopez Luna, Stefanie Kühl, Uta Hejral, Earl M. Davis, See Wee Chee, Janis Timoshenko, Duncan T.L. Alexander, Arno Bergmann, Beatriz Roldan Cuenya

**Affiliations:** a Department of Interface Science, Fritz-Haber-Institute of the Max-Planck Society Faradayweg 4-6 14195 Berlin Germany roldan@fhi-berlin.mpg.de; b Electron Spectrometry and Microscopy Laboratory (LSME), Institute of Physics (IPHYS), École Polytechnique Fédérale de Lausanne (EPFL) Lausanne CH-1015 Switzerland

## Abstract

Electrochemical reduction of CO_2_ (CO_2_RR) is an attractive technology to reintegrate the anthropogenic CO_2_ back into the carbon cycle driven by a suitable catalyst. This study employs highly efficient multi-carbon (C_2+_) producing Cu_2_O nanocubes (NCs) decorated with CO-selective Au nanoparticles (NPs) to investigate the correlation between a high CO surface concentration microenvironment and the catalytic performance. Structure, morphology and near-surface composition are studied *via operando* X-ray absorption spectroscopy and surface-enhanced Raman spectroscopy, *operando* high-energy X-ray diffraction as well as quasi *in situ* X-ray photoelectron spectroscopy. These *operando* studies show the continuous evolution of the local structure and chemical environment of our catalysts during reaction conditions. Along with its alloy formation, a CO-rich microenvironment as well as weakened average CO binding on the catalyst surface during CO_2_RR is detected. Linking these findings to the catalytic function, a complex compositional interplay between Au and Cu is revealed in which higher Au loadings primarily facilitate CO formation. Nonetheless, the strongest improvement in C_2+_ formation appears for the lowest Au loadings, suggesting a beneficial role of the Au–Cu atomic interaction for the catalytic function in CO_2_RR. This study highlights the importance of site engineering and *operando* investigations to unveil the electrocatalyst's adaptations to the reaction conditions, which is a prerequisite to understand its catalytic behavior.

## Abstract 2

Adding small amounts of Au Nanoparticles on Cu_2_O Nanocubes boost the selectivity of C_2+_ products during electrocatalytic CO_2_ reduction reaction.

## Graphical abstract

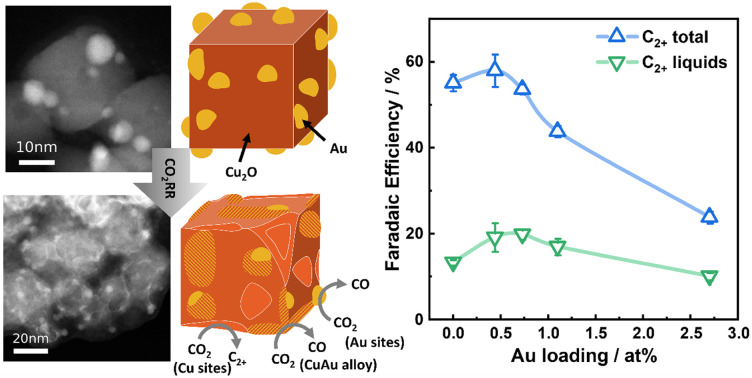

**Broader context**

Environmentally friendly technologies for storage and reutilization of energy are important to maintain a sustainable carbon cycle. Here, the electrocatalytic reduction of CO_2_ attracts by recycling CO_2_ with renewable energy to form highly valued chemicals. By using Cu-based materials as catalysts, the performance of the CO_2_ reduction reaction can be tuned towards C_2+_ products, such as ethanol or ethylene. However, major challenges are the in-depth understanding of reaction mechanism, including the performance towards selective products, as well as creating stable catalysts. Thus, careful characterization of the catalysts is necessary to understand how the catalysts evolve during the applied reaction. Herein, we studied a bimetallic Cu-based material by adding increasing amounts of CO-forming Au nanoparticles to understand the structural, compositional and morphological changes under reaction conditions, correlating them with the obtained selectivities and thus extracting structure–selectivity relationships. The results from this work highlight the essential changes and requirements for Cu–Au based catalysts that are relevant to create high-performing catalysts for the electrocatalytic CO_2_ reduction reaction.

## Introduction

The electrochemical reduction of CO_2_ (CO_2_RR) is an attractive technology for closing the anthropogenic carbon cycle by using renewable energy such as solar- or wind-power to convert the greenhouse gas CO_2_ into energy-dense feedstock chemicals or liquid fuels.^1,2^

The ideal electrocatalysts for this reaction require low overpotentials, high stability and excellent selectivity for C_2+_ products while minimizing the parasitic hydrogen evolution reaction (HER). Whereas various materials may reduce CO_2_ into C_1_ products (CO, CH_4_, HCOOH), copper-based materials are unique in producing C_2+_ hydrocarbons (*e.g.*, C_2_H_4_), alcohols (*e.g.*, C_2_H_5_OH) or carbonyls, due to their ability to enable the C–C coupling. Cu_2_O nanocubes (NCs) are reported as one of best catalysts for C_2+_ products.^3^ The Cu-based catalysts owe their good selectivity to an optimal binding and stabilization of the CO intermediates^4^*e.g.*, *CO and *CO_2_^−^). However, these catalysts still suffer from low energy efficiency and lack of selectivity toward a specific reaction product.^5^

Among the multitude of approaches to improve the selectivity towards C_2+_ products, sequential catalysis has been proposed for optimizing the selectivity by combining both, beneficial electronic effects *via* alloying and an increased CO coverage.^6–10^ By adjusting the local electronic structure of the catalyst though the use of bimetallic systems and alloys, the binding energies of the reaction intermediates such as *CO might be altered and thus, the reaction pathways might be modified.^11–14^ Furthermore, a higher local concentration and a subsequently higher coverage of adsorbed CO molecules on Cu can be induced through CO-selective co-catalysts, such as Au, Ag or Zn.^4^ This may lead to subsequent C–C coupling and thus, enhanced selectivity for C_2+_ products.^15–17^ Despite their similar abilities to efficiently reduce CO_2_ to CO, Au, Ag and Zn differ from each other in the onset potential for CO production with −0.25 V (*vs.* RHE) for Au, −0.52 V for Ag and −0.6 V for Zn.^4^ Thus, Au has the lowest overpotential and shows the highest CO partial current density, which can be explained by its weak *CO binding strength.^4,18,19^ Furthermore, it has been proposed that the thus produced CO might follow a CO spillover mechanism, which has also been reported for Ag/Cu catalysts.^20,21^

Moreover, Au is an attractive co-catalyst for CO_2_RR due to its particular alloying tendency with Cu, which allows tuning the electronic structure better than the less miscible Ag–Cu catalysts.^18,22^ In particular, CuAu alloys can form three ordered phases with different compositions (Cu_3_Au, CuAu, CuAu_3_),^23^ and CuAu superstructures^24^ as well as unordered phases, which present a wide variety of possible active species for CO_2_RR. CuAu alloyed systems have been reported to lead to enhanced CO_2_RR performance, which was attributed to different factors, including a possible protection from the formation of Cu oxide;^22,25^ an increased formation of CO, paralleled by the suppression of HER and CH_4_ formation;^26–28^ a shift in the onset potential for CO_2_RR towards lower overpotentials;^22,29^ *COOH stabilization^12,30,31^ and to synergistic geometric and electronic effects that boost C_2+_ production toward alcohols.^32–36^ However, the type and the influence of the alloyed structure under CO_2_RR conditions as well as the high CO coverage on Cu through CO producing co-catalysts remains an open question. Additionally, a systematic study of CuAu NP catalysts with a complex mixing pattern of the constituent active elements, alloy formation and the interfaces of its multiple phases under *operando* conditions has not yet been reported. These insights are critically needed to enable true catalyst design based on comprehensive scientific understanding of catalyst adaptations under reaction conditions and ensuring sustainable utilization such as noble metals co-catalysts.

Hence, in this work we use Cu_2_O nanocubes (NC) decorated with varying amounts of Au NPs as tandem catalysts to reveal the role of alloying and of the CO coverage on the product selectivity by analyzing the alloy formation and the influence of CO-rich micro-environments under CO_2_RR conditions. The compositional restructuring was investigated with scanning transmission electron microscopy and X-ray photoemission spectroscopy, while the active phases under reaction conditions were deconvoluted with *operando* high energy X-ray diffraction, *operando* X-ray absorption fine structure spectroscopy, and quasi *in situ* X-ray photoelectron spectroscopy. The CO coverage on Cu was followed using *operando* surface-enhanced Raman spectroscopy. Correlations between the alloy type and its evolution during CO_2_RR as well as the CO coverage and the product distributions were drawn, unveiling that optimized alloyed phases and CO coverages result in increased C_2+_ product selectivity.

## Results and discussion

**Fig. 1:**
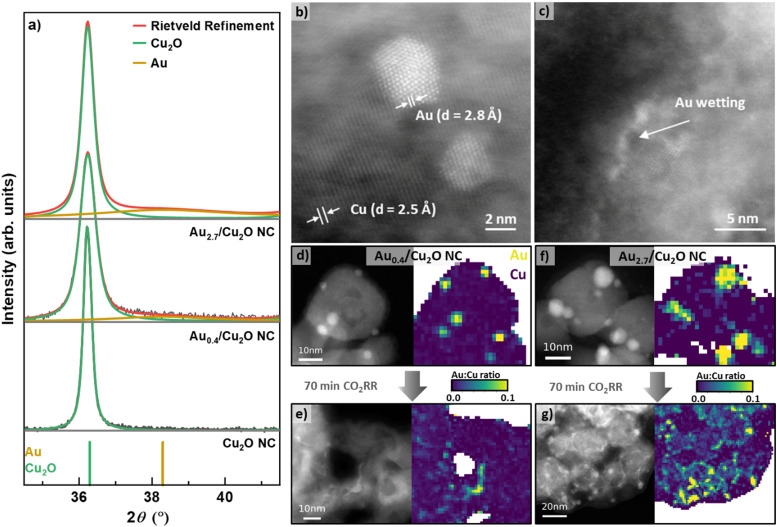
(a) *Ex situ* XRD patterns of the Cu_2_O NCs, Au_0.4_/Cu_2_O NCs and Au_2.7_/Cu_2_O NCs and their corresponding Rietveld refinement fits. STEM-HAADF images of (b) Au monocrystalline and polycrystalline NPs on the surface of the Au_0.4_/Cu_2_O NCs in their as-prepared state and (c) an example of Au wetting on the surface of the same Au_0.4_/Cu_2_O NCs after 70 min CO_2_RR. STEM-HAADF images with corresponding EDXS mappings of (d) and (e) Au_0.4_/Cu_2_O NCs and (f) and (g) Au_2.7_/Cu_2_O NCs in the as-prepared state (d) and (f) and after 70 min CO_2_RR (e) and (g). The reaction was carried out in CO_2_-saturated 0.1 M KHCO_3_ at −1.05 *V*_RHE_.

We prepared Cu_2_O NCs decorated with different Au NP loadings and applied scanning transmission electron microscopy (STEM) imaging using the high angle annular dark field (HAADF) detector to verify the cubic shape of the as-prepared Cu_2_O NCs. With Au loading, the cubic shape of the as-prepared catalysts remains recognizable, but the corners become progressively rounded as the Au loading increases, Fig. S1 (ESI†). The Au NPs are observed to be evenly dispersed on the surfaces of the NCs, for all loadings from lowest (Au_0.4_/Cu_2_O NCs) to highest (Au_2.7_/Cu_2_O NCs). The lower limit of Au loading was set by the decrease in the homogeneity of the Au NP decoration on the surface of the Cu_2_O NCs. Interestingly, using atomic resolution aberration-corrected HAADF STEM, the Au NPs are observed to exhibit both single grain and multigrain structures, Fig. 1b.

Using Inductively Coupled Plasma–Mass Spectrometry (ICP–MS), we determined the bulk compositions of Au with varying loadings on the Cu_2_O NCs to be 0.43 at%, 0.77 at%, 1.08 at%, and 2.69 at% for Au_0.4_/Cu_2_O NC, Au_0.8_/Cu_2_O NC, Au_1.1_/Cu_2_O NC and Au_2.7_/Cu_2_O NC, respectively, Table S1 (ESI†). X-ray diffraction (XRD) analysis of the as-prepared NCs demonstrates the presence of Cu_2_O, with its most intense reflections (111) at 36.4° and (200) at 42.3°, Fig. 1a and Fig. S2 (ESI†). The addition of the Au NPs induces broad and weak Au(111) reflections at 37.34°, which are present with increasing intensity for all catalysts with increasing Au loading. The coherence lengths obtained by Rietveld refinement for the Cu_2_O NCs of all catalysts vary between 21 and 42 nm, Table S2 (ESI†). These findings are in agreement with the Cu_2_O NC edge length and Au NP size distributions displayed in Fig. 1d, f and Fig. S3 and Table S3 (ESI†), revealing cube edge lengths of *ca.* 20 nm and Au NPs sizes of 3–4 nm. We furthermore characterized the oxidation behavior of our catalysts by cyclic voltammetry (CV), Supplementary Note 2, Fig. S13 and Table S6 (ESI†).

The evolution of the catalysts under CO_2_RR conditions was studied to understand the catalyst function and to correlate the findings with the product selectivity. Therefore, we carefully analyzed STEM images, together with STEM energy dispersive X-ray spectroscopy (EDXS) elemental maps, of samples before and after CO_2_RR to identify any irreversible morphological restructuring and Au wetting effects on the Cu surface. Fig. 1 and Fig. S4–S7 (ESI†) depict the HAADF-STEM images (including aberration-corrected) and the EDXS elemental and ratio maps of the catalysts before and after CO_2_RR. Note that the EDXS maps are spatially binned in order to improve signal to noise ratio for their quantification. Two selected catalysts show well-dispersed Au NPs on the surface of the Cu_2_O NCs in the as-prepared state, Fig. 1d, f and Fig. S7 (ESI†). After 70 min of CO_2_RR at −1.05 V, strong morphological changes are observed for the Au–Cu_2_O NCs, losing their cubic shape and appearing porous, Fig. S5–S7 (ESI†). Note here that the catalysts in the present STEM study have undergone exposure to air before the microscopic analysis, but the results are in line with recent *in situ* TEM studies in which the shape of the pure Cu_2_O NCs was investigated during CO_2_RR.^37^ The NC edge lengths and the Au NP sizes did not change upon CO_2_RR as compared to the as-prepared catalysts within the experimental uncertainty. It is noteworthy that the morphological changes of the Au_0.4_/Cu_2_O NCs after CO_2_RR resemble the hollow CuOx frames that have been previously observed for pure Cu_2_O NCs.^9,38^ The other catalysts exhibit morphological changes dominated by the surface Au coverage such as the density of Au NPs decreased and Au was incorporated into Cu as fine stripes in between the retained Au NPs, Fig. 1e, g and Fig. S7 (ESI†). This Au wetting leads to a skeleton-like structure in which Cu reshapes into a new frame. A more detailed analysis on the wetting effect of the Au_2.7_/Cu_2_O NCs was performed *via* aberration-corrected STEM, Fig. S4 and S5 (ESI†). The images clearly show separate phases in the as-prepared state and an Au-wetted Cu surface after CO_2_RR framing around the nanostructures, Fig. 1b and c. This Au wetting phenomenon is more pronounced for the higher Au loadings, rearranging the catalyst structure towards an Au-rich frame, Fig. 1e, g and Fig. S7 (ESI†). Indeed, significant changes in the Cu morphology were found for the low Au loading catalysts, partially with coalescence and dissolved Cu. On the other hand, when higher Au loadings on the Cu_2_O catalysts are considered, a framed-like shape is more easily preserved, Fig. S7 (ESI†). However, for the Au_2.7_/Cu_2_O NCs, even after 1 h of CO_2_RR there are still Au NPs preserved, suggesting that the Au is not completely alloyed in this sample, which increases the compositional complexity of the catalysts, with different Au and Cu-rich phases being present during catalysis.

We also followed the changes in the composition by quantifying spatially integrated EDX spectra from the EDXS maps, Table S4 (ESI†), and found the expected increased Au : Cu ratio trend with increasing nominal Au loading, though with a slight offset as compared to the results obtained by ICP–MS, Table S5 and Fig. S8 (ESI†). Note that EDXS is a semiquantitative method without external standards; while Au : Cu ratios show a definitive trend, the obtained values should not be treated as absolutes. The Au : Cu ratio was also locally mapped with the spatially-binned EDXS data, Fig. 1d–g and Fig. S7 (ESI†). Thus, for the as-prepared catalysts, defined NPs are observed, while after CO_2_RR, both distorted Au NPs and Au wetting of the Cu surfaces were observed. The Au wetting and skeleton-like structures are clearly visible.

**Fig. 2:**
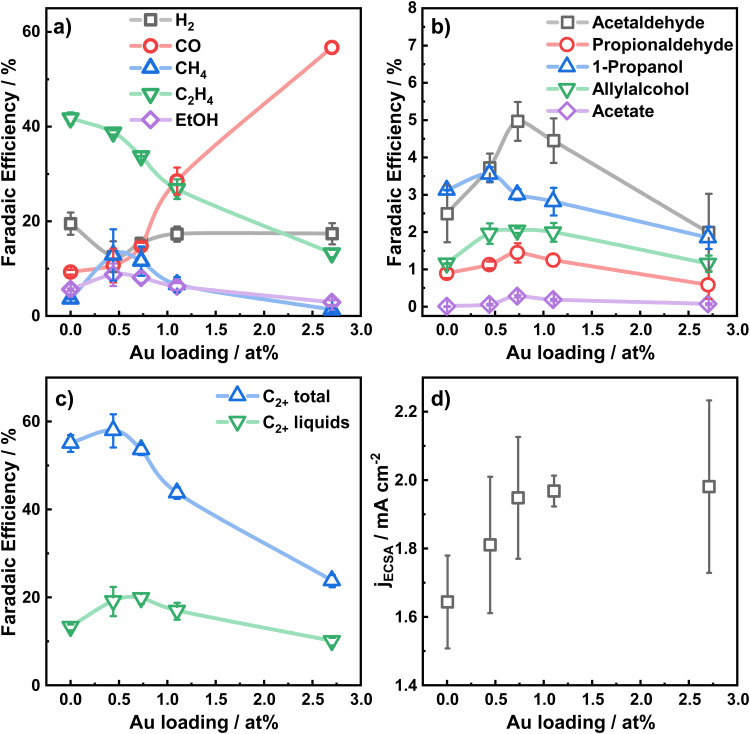
Faradaic efficiencies at −1.07 V in 0.1 M KHCO_3_ as a function of the Au NP loading for (a) H_2_, CO, CH_4_, C_2_H_4_, (b) minor liquid products, (c) C_2+_ total products, C_2+_ liquid products; (d) Au-loading dependency of the current density normalized by the electrochemical surface area.

The electrocatalytic performance of the catalysts was evaluated by chronoamperometric measurements at −1.07 V for 1 h in CO_2_-saturated 0.1 M KHCO_3_ for the different Au loadings. Fig. 2a–c shows the selectivity trends in form of faradaic efficiencies (FE) as a function of the Au loading and the corresponding current densities (*d*). The potential-dependent FEs and corresponding currents for each catalyst are given in Fig. S9 (ESI†). In Fig. 2b, the increased formation of CO with increasing Au loading up to a FE of 56% for Au_2.7_/Cu_2_O NC is evident.

This high production of CO on Au suggests a high local CO concentration around the Au NP perimeter in the proximity of Cu. In return, the C_2_H_4_ production is indirectly correlated to the CO production, sacrificing the FE of C_2_H_4_ for CO for large Au coverages on the Cu_2_O NCs. Hydrogen production decreases for the lower Au loadings (<1%), while a slightly higher CH_4_ production is found. The FE of ethanol increases slightly by 2 percentage points for Au_0.4_/Cu_2_O NCs and Au_0.8_/Cu_2_O NCs. Remarkably, the FEs of the minor liquid products such as propionaldehyde, acetaldehyde and allylalcohol are also boosted for the lowest Au loadings. Acetaldehyde, which is considered as a possible precursor of ethanol,^39^ is improved for all Au loadings investigated and is highest for Au_0.8_/Cu_2_O NC, with 4.8% FE, 2.6 times higher than for the pure Cu_2_O NCs. For this catalyst, the highest FE is also observed for propionaldehyde, while allylalcohol has its peak FE for the three low Au loading catalysts and 1-propanol is the highest for Au_0.4_/Cu_2_O NC.

For the bimetallic catalysts, the increased ethanol production is typically linked to a weaker binding strength of the *CO intermediates to the secondary catalysts and to a CO spillover mechanism, which is described well for Ag–Cu systems.^5,9,40,41^ Although Au–Cu systems tend to alloy under reaction conditions, the CO spillover mechanism for these systems is not clearly understood. In our case, and in contrast to the similarly prepared Ag–Cu_2_O NCs,^9^ the addition of Au as co-catalyst does not improve the ethanol production significantly, despite the increased production of acetaldehyde. Ethanol is understood to be produced either directly from the *CH_2_CHO intermediate through protonation, or in small parts also through re-adsorption of acetaldehyde (CH_3_CHO) with subsequent protonation.^39^ We explain the preferred production of acetaldehyde and the impeded production of ethanol by the CO-richer environment that hinders the protonation of the *CH_3_CHO intermediate and the re-adsorption of acetaldehyde on the catalyst.

The lowered hydrogen production, observed for our Au_*x*_/Cu_2_O NC catalysts, is in agreement with impeded *H coverage on the surface and OH^−^ formation.

Fig. 2c displays the total C_2+_ product FE, which is the highest for Au_0.4_/Cu_2_O NC and exceeds slightly the ones of Cu_2_O NC and Au_0.8_/Cu_2_O NCs. With increasing Au loading, the C_2+_ product formation reduces drastically, which results mainly from the decreased FE of ethylene and ethanol. The C_2+_ liquid product formation for the two lowest Au loading catalysts exceeds that of the pure Cu_2_O NCs by up to 5% FE. The C_2+_ carbonyl formation, however, increases for the low Au loadings and decreases drastically for the high Au loadings, demonstrating the beneficial effect of low amounts of Au on Cu_2_O NCs for CO_2_RR. In a similar way, the FE of the liquid C_2+_ products for the low Au loadings increases to a maximum of 21% FE and decreases for the Au_2.7_/Cu_2_O NCs. The combined liquid products follow the same trend, Fig. 2c. Thus, a switch in the selectivity for the gaseous products from C_2_H_4_ to CO takes place, and for the Au_2.7_/Cu_2_O NCs the CO selectivity is so high that fewer liquid products are formed.

The total and partial current densities at −1.07 V *vs.* RHE, normalized by the electrochemical surface area (ECSA) are shown as a function of the Au loading in Fig. 2d and Fig. S10 (ESI†) and represent the intrinsic catalytic turnover. The total current density, Fig. 2d, increases slightly with increasing Au loading, indicating a higher activity of Au compared to Cu, and consequently higher CO production rate, in accordance with literature.^31^ Furthermore, the partial current densities for C_2+_ products, Fig. S10 (ESI†), are the highest for the low Au loadings, with decreasing activities for increasing Au loadings. Notably, the lowest Au loading of 0.4 at% leads to a 2-fold increase in C_2+_ current density and a 4-fold increase for the C_2+_ liquid product current density compared to pure Cu_2_O NCs. Thus, the turnover of CO to C_2+_ products appears to be sensitive to the presence of small amounts of Au (low loadings), leading to a slightly CO-richer environment, while higher Au loadings and thus, increased CO formation, are detrimental.

Stability tests over 20 h on all catalysts confirm stable product distributions after the initial alloy formation during the first hour and are further described in Supplementary Note 1 and Fig. S11 and S12 (ESI†).

**Fig. 3:**
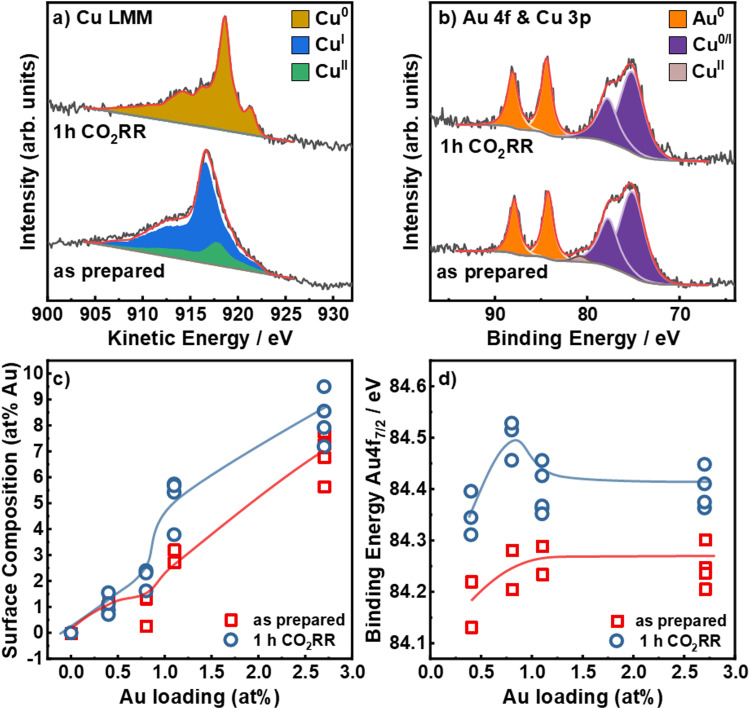
Quasi *in situ* XPS spectra for the Au_2.7_/Cu_2_O NCs: (a) Cu LMM Auger and (b) Au 4f and Cu 3p in the as-prepared state and after 1 h CO_2_RR at −1.05 V *vs.* RHE in 0.1 M KHCO_3_. (c) Au : Cu ratio (at%) at the catalysts surface in the as prepared state and after CO_2_RR as a function of the nominal Au loading (determined by ICP–MS). (d) Binding Energies of the Au 4f_7/2_ core level region in the as-prepared state and after 1 h CO_2_RR.

To understand the irreversible changes of the surface composition, chemical state and alloy formation of the bimetallic catalysts upon CO_2_RR, we employed quasi *in situ* and X-ray photoemission spectroscopy (XPS). In comparison to *ex situ* measurements, this avoids post-reaction air exposure of the catalysts that may induce surface re-oxidation and possible restructuring. Fig. 3 shows the Cu L_3_M_4,5_M_4,5_ Auger electron spectra (a) and the Au 4f and Cu 3p (b) core level XPS regions for the Au_2.7_/Cu_2_O NCs before and after 1 h CO_2_RR at −1.05 V, as well as the surface composition (c) and the binding energy of the Au 4f_7/2_ peak (d) as a function of the nominal Au loading. The Cu 2p, Au 4f, Cu^38^ 3p XPS and Cu LMM XAES for all catalysts are shown in Fig. S14–S16 (ESI†). The Cu LMM Auger spectra in Fig. 3a show a combination of Cu^0^, Cu^l^ and Cu^ll^ in the as-prepared state and a reduction of Cu^ll^/Cu^l^ to Cu^0^ after CO_2_RR. In the as-prepared states, the catalysts present similar ratios of Cu_2_O (80–90%) and CuO (10–20%), whereas, after 1 h under CO_2_RR at −1.05 V, all catalysts are reduced to metallic Cu within the error margins, Fig. S14, S15 and Table S7 (ESI†). The Au 4f spectra in Fig. 3b show peaks at *E*_BE,7/2_ = 84.2 eV and *E*_BE,5/2_ = 87.9 eV. This increased binding energy of +0.2 eV compared to metallic Au (84.0 eV) can be assigned to alloy formation or to reduced interfacial charge transfer between Cu_2_O and Au.^42^

The surface composition of the catalysts was determined by comparing the Cu 3p and Au 4f areas, Fig. 3b, c and Table S8 (ESI†). In the as-prepared state, the Au surface loadings are with ∼1 to ∼6% notably higher than the average bulk composition. The Au surface composition increased after 1 h CO_2_RR for the higher Au loadings. This effect can be assigned to a combination of dynamic redistribution, segregation and wetting of Au on the Cu surface. These findings agree well with the STEM-EDXS and ICP–MS data presented in the supporting information, Tables S4 and S5 (ESI†).

Fig. 3d demonstrates that the Au 4f binding energy does not vary strongly with Au loadings in the as-prepared state. However, the Au 4f binding energy increased upon CO_2_RR by 0.2 eV, suggesting a change of the electronic structure around the Au NPs and the possible formation of an alloy between Au and Cu.^43^ However, this change could also include the contribution of a charge transfer effect, which would similarly lead to higher binding energy shifts, as detected for the catalysts in the as-prepared state. Furthermore, depending on the Au loading, the binding energy after CO_2_RR varies from 84.3 eV to 84.4 eV. Remarkably, the Au_0.8_/Cu_2_O NCs show relatively high binding energies of the Au 4f_7/2_, indicating higher alloy formation and also possibly the presence of sample regions containing pure Au clusters or small NPs which are also characterized by large positive binding energy shifts. This observation coincides with a lower CO, C_2+_ liquid products and H_2_ specific activities, Fig. S10 (ESI†), as compared to the Au_0.4_/Cu_2_O NCs. Our findings suggest that a large content of CuAu alloy formation is detrimental for C_2+_ production from CO_2_RR. As we expected to have a large fraction of CuAu alloy regions with increasing Au loading after CO_2_RR, we conclude that the shift in binding energy detected must be the convolution of the regions with CuAu alloys and those with very small pure Au clusters of NPs. It is plausible that in some of our samples, like Au_0.8_/Cu_2_O NCs there exists a larger compositional heterogeneity combining CuAu alloyed regions and low-coordinated Au regions. Overall, our data also reveal a significant decrease in the density of pristine Cu sites during CO_2_RR.

To extract comprehensive information on the catalyst's bulk structure during CO_2_RR, we employed *operando* high energy XRD (HE-XRD) to understand the formation of crystalline Cu_1−*x*_Au_*x*_ alloy phases during CO_2_RR. Note that highly disordered metallic or cationic phases cannot be detected using XRD. We have previously shown that bare Cu_2_O NCs mostly reduce to metallic Cu using operando HE-XRD.^44^ Fig. S17 (ESI†) presents the HE-XRD pattern of the Cu_2_O NCs recorded at 67 keV at open circuit potential (OCP) and after 1 h at −1.05 V. For the Au_*x*_/Cu_2_O NCs, at OCP, the diffraction pattern agrees well with the Cu_2_O phase, in addition to the strong background caused by the electrolyte. During CO_2_RR, the Cu_2_O Bragg peaks disappear almost completely, and a diffraction pattern of metallic Cu develops, in which the broad feature at ∼4.65° could be caused by Cu_1−*x*_Au_*x*_ phase(s). Compared to the as-prepared Au_2.7_/Cu_2_O NCs at OCP, the Bragg peaks of metallic Cu of Au_2.7_/Cu_2_O NCs during CO_2_RR are significantly broader, suggesting a shorter structural coherence length. Rietveld refinement reveals a coherence length of ∼7 nm of the metallic Cu domains compared to ∼17 nm for the Cu_2_O domain at OCP. The structural properties of the potential Cu_1−*x*_Au_*x*_ minority phase were not reliably resolvable using Rietveld refinement. The Cu lattice parameter of ∼3.6499 Å did not show any evidence for Au incorporation into the main Cu phase during CO_2_RR. Thus, *operando* HE-XRD shows the reduction of the Au_2.7_/Cu_2_O NCs during CO_2_RR, which consists mainly of a bulk Cu phase with a potential highly disordered Cu_1−*x*_Au_*x*_ minority phase on the surface. Further details are given in Table S9 (ESI†).

**Fig. 4:**
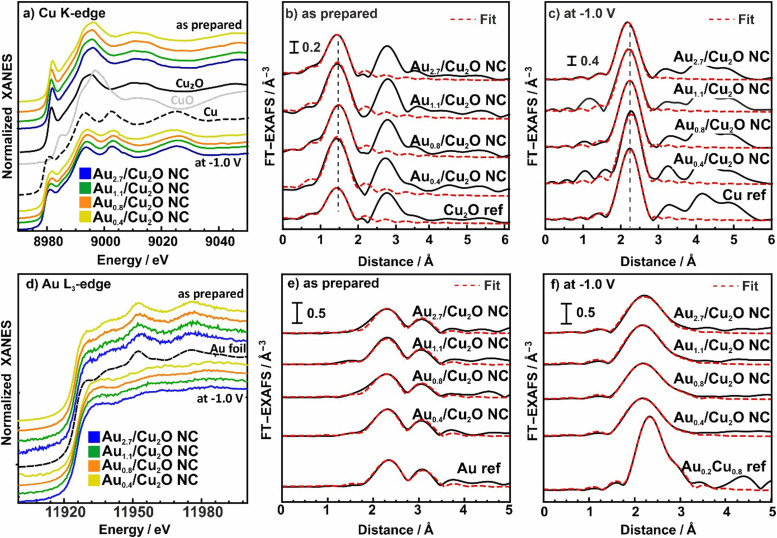
Normalized XANES spectra from (a) the Cu K-edge and (d) Au-L_3_ edge of bimetallic AuCu NCs with different Au : Cu ratios measured as-prepared and under CO_2_RR at −1 *V*_RHE_, plotted together with bulk Cu, Cu_2_O and CuO references. FT- EXAFS data from the Cu K-edge (b) and (c) and Au-L_3_ edge (e) and (f) are also shown together with the corresponding fits for the as-prepared samples (b) and (e) and those during 4 h of CO_2_RR (c) and (f). Reference EXAFS spectra of an Au foil and a Cu_0.2_Au_0.8_ reference alloy are shown for comparison. Cu_0.2_Au_0.8_ reference was redrawn from ref. 46.

To understand the chemical composition, interatomic interactions and alloy formation during CO_2_RR were extracted from *operando* X-ray absorption spectroscopy (XAS). This technique is highly complementary to the XRD data presented above, since it unveils the disordered phases present in these samples under the different environments and reaction conditions. The analysis of the Cu K-edge X-ray absorption near edge structure (XANES) and extended X-ray absorption fine structure (EXAFS) data is shown in Fig. 4a–c and Fig. S18, S19 and Table S10 (ESI†).

Fig. 4a shows the XANES of the Cu K-edge for the samples in the as-prepared state and during CO_2_RR in steady state at 4 h CO_2_RR at −1.0 V. The XANES spectra for as-prepared catalysts demonstrate the presence of oxidized Cu species, mostly in the Cu^l^ state, as suggested by the characteristic pre-edge feature. Nonetheless, significant amounts of Cu are also oxidized to Cu^ll^. After 4 h of CO_2_RR, the Cu of all catalysts is mostly reduced to Cu^0^. These results are in agreement with the previously discussed XPS data.

The evolution of the local atomic structure around Cu under CO_2_RR was tracked by studying the Fourier transformed (FT-EXAFS) spectra of the Cu K-edge, Fig. 4b, c and Table S10 (ESI†). In the as-prepared state, Cu K-edge FT-EXAFS exhibits prominent peaks at 1.85 Å and 2.8 Å (phase-uncorrected) corresponding to Cu–O and Cu–Cu bonds in bulk Cu_2_O-like structure. Data fitting revealed that the coordination number of the Cu–O bonds are 3.0–3.6, which is higher than the one for Cu_2_O (2), indicating significant amounts of CuO in our catalysts. Under CO_2_RR, the FT-EXAFS features corresponding to Cu oxide decrease while a new peak corresponding to the Cu–Cu distances in metallic Cu appears at 2.5 Å. Data fitting suggest that the corresponding Cu–Cu coordination number after reaching the equilibrium state after 4 h is close to 12, which fits well to the fcc structure of metallic Cu.^45^ Note here that no significant contribution of Cu–Au bonds to Cu K-edge FT-EXAFS data are observed, due to the low Au-to-Cu ratio of the catalysts.

The corresponding Au L_3_-edge XANES data in Fig. 4d demonstrate that the Au NPs in the as-prepared catalysts are in a metallic state, with a local environment similar to that in the Au foil reference material. However, during CO_2_RR, a white line feature at 11930 eV appears, reflecting a more cationic character of the Au atoms, and thus, changes in the electronic structure due to alloying with Cu and an accompanying charge transfer to the Cu.^43,47^ Moreover, we observe significant changes in the post-edge features, suggesting strong differences in the interatomic distances and/or changes in the types of nearest neighbors of the absorbing Au atoms as compared to bulk Au. We note that the evolution of the Au L_3_-edge XANES spectra proceeds similarly for all our catalysts, regardless of the Au loading.

We obtained further insights into the local atomic structure of the Au atoms from the fitting of the FT-EXAFS spectra, Fig. 4e, f and Table S11 (ESI†). The as-prepared catalysts exhibit a prominent peak at 2.3 Å (phase uncorrected), resembling Au–Au bonds with a distance *R*_Au−Au_ of 2.8 Å and a coordination number (CN) of around 12(2), matching the results obtained for the Au foil (*R*_Au−Au_ 2.9 Å, CN = 12). This is analogous to the conclusions extracted from the XANES spectra as well as with the TEM and XPS data showing Au NPs that are well dispersed and attached to the Cu_2_O NC surfaces. During CO_2_RR, the Au–Au coordination number decreases, while the Au–Cu contribution evolves with coordination numbers between 8 and 10. Interestingly, we observe a significant mismatch between Au–Au and Au–Cu bond lengths for all catalysts, where the former remains similar to that in the Au foil (2.86 Å), while the latter is *ca.* 2.62 Å, which is clearly larger than the Cu–Cu bond lengths in bulk metallic Cu (2.54 Å).^45^ These results suggest a heterogeneous structural evolution in which Au-rich regions coexist with regions of a Cu-rich CuAu alloy. The average interatomic distance for Au–Cu of 2.62 Å agrees best with a 1 : 1 AuCu-like phase, following Vegard's rule. Furthermore, we note that the CN ratio of the Au–Au and Au–Cu distances during CO_2_RR seems to decrease with increasing Au content for loadings above 0.4 at%. Thus, our *operando* analysis shows that for all Au loadings a CuAu alloy of low crystallinity and/or domain size forms during CO_2_RR. This conclusion is compatible with the Au wetting behavior observed with STEM and EDXS analysis.

**Fig. 5:**
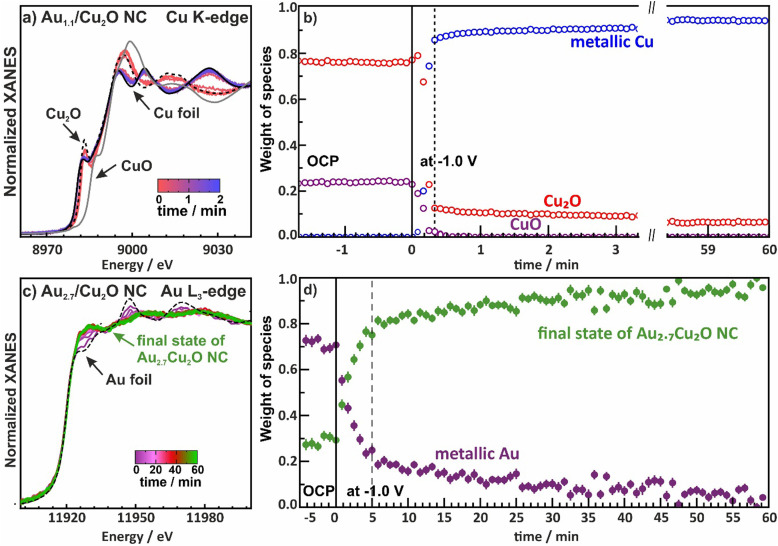
(a) Normalized Cu K-edge XANES spectra and (b) weight of the observed species as a function of time during CO_2_RR at −1.0 V for the Au_1.1_/Cu_2_O NCs as an example. (c) Normalized Au L_3_-edge XANES spectra and (d) weight of the species as a function of time during CO_2_RR at −1.0 V unveiling the formation of an CuAu alloy for the Au_2.7_/Cu_2_O NCs as example. The final state of Au_2.7_/Cu_2_O NCs is a mixed state with Cu-rich, Au-rich and CuAu alloy phases.

Furthermore, we investigated the time evolution of the species from both the Cu and Au perspective over 60 min by collecting XAS spectra every 1 s for Au_2.7_/Cu_2_O NC (in QXAS mode) and every 12 min for Au_0.4_/Cu_2_O NC and Au_0.8_/Cu_2_O NC, Fig. 5 and Fig. S19 and S20 (ESI†). The chemical state components were quantified *via* linear combination analysis (LCA) using reference spectra. For the Cu-K edge, the initial states in the electrolyte show varying contributions of Cu_2_O and CuO, which might be attributed to an aging effect of the catalysts and/or a beam damage effect, Fig. 5a, b and Fig. S19 (ESI†). Upon application of −1.0 V, while the majority of the Cu_2_O reduces within 20 s to metallic Cu, after 4 h of CO_2_RR fractions of 10% Cu_2_O are nevertheless observed. Given that quasi *in situ* XPS showed a purely metallic catalyst surface after 1 h CO_2_RR, this suggests that a robust Cu_2_O phase remains in the core of the catalysts. These remnant Cu oxide species even after prolonged exposure to CO_2_RR conditions were also observed for analogous pure Cu_2_O and Ag-decorated Cu_2_O nanocubes.^9,44^ Meanwhile, the Cu–Cu coordination numbers during CO_2_RR suggest a bulk-like metallic Cu as the dominant phase, which is in agreement with the insights from *operando* HE-XRD. Interestingly, the CuO of the Au_2.7_/Cu_2_O catalysts first reduces to Cu_2_O as the Cu_2_O LCA weight first increases by about 5–10 percentage points before the catalysts reduces further, which is also in accord with previous observations.^44^

We then evaluated the time evolution of the Au L_3_-edge and show the weight of the different species as a function of time with a final state of the Au_2.7_/Cu_2_O NC as reference for the alloy phase, Fig. 5c, d and Fig. S20 (ESI†). It should be noted that the chosen final state of the Au_2.7_/Cu_2_O NC is a mixture of Cu-rich, Au-rich and CuAu-alloy phases. The local structure around Au evolves much slower than the reduction of Cu, with about one hour needed to reach a stable alloy phase, where the alloy formation is not evolving significantly anymore. Thus, these results might explain the slow product adaptation of the Au-containing catalysts in the long-term CO_2_RR measurements over 20 h. Restructuring of the catalyst during alloy formation represents significant atom mobility and continuous altering of the active sites which in turn continuously affects the product distribution.

**Fig. 6:**
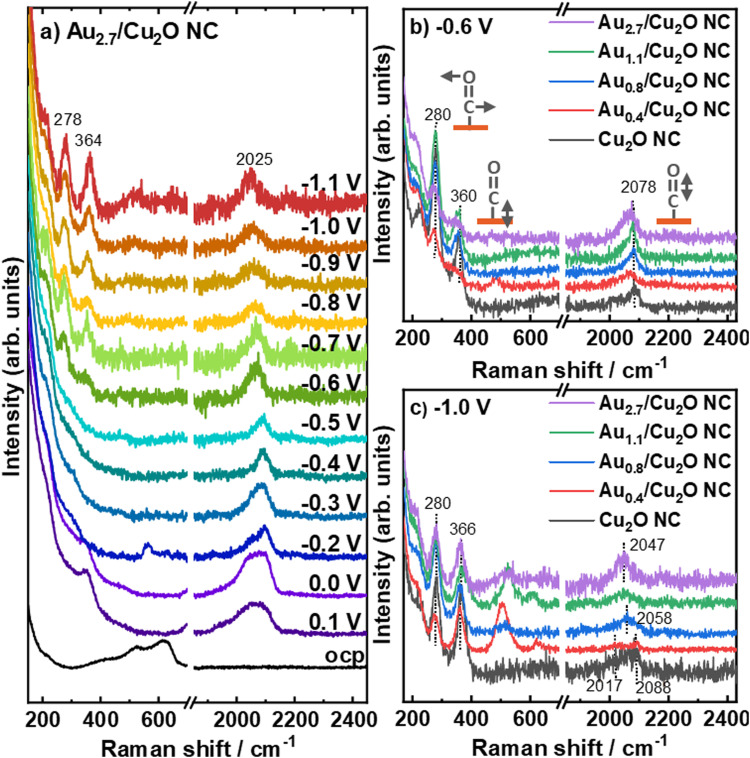
*Operando* SERS of (a) Au_2.7_/Cu_2_O NC under potential dependence and of all Au–Cu_2_O catalysts at (b) −0.6 V and (c) −1.1 V in CO_2_-saturated 0.1 M KHCO_3_. Data for the Cu_2_O NC are redrawn from ref. 9.

We furthermore study the role of the potential-dependent CO-related adsorbates with varying Au loading by using *operando* surface-enhanced Raman spectroscopy (SERS), Fig. 6 and Fig. S21, S22 (ESI†). Fig. 6a shows the SERS data recorded at potentials between OCP and −1.1 V *vs.* RHE for the Au_2.7_/Cu_2_O NCs, as an example for all investigated catalysts (Fig. S22, ESI†). At OCP, the Raman scattering of the Cu_2_O exhibits the typical bands at 415 cm^−1^ (multiphonon process), 527 cm^−1^ (Raman active F_2g_ vibrational mode), 623 cm^−1^ (IR active F_1u_ mode) and 220 cm^−1^ (overtone 2E_u_). These bands were found to disappear at 0.2 V for all catalysts, which reflects the electrochemical reduction of Cu_2_O to Cu at the surface.^48^ Between 0.2 V and 0.1 V, bands at 360 cm^−1^ appear with corresponding bands at 706 cm^−1^, 1050 cm^−1^ and 1074 cm^−1^, which can been assigned to surface copper carbonate or bidentate carbonate species, Fig. S22 (ESI†).^38,49^ Remarkably, a CO stretching band around 2090 cm^−1^ was observed at 0.2 V for the three higher Au loadings and may be linked to carbonates or *H*_ad_. These CO stretching bands around 2090 cm^−1^ are significantly stronger for the Au_2.7_/Cu_2_O NCs compared to the Au_0.8_/Cu_2_O NCs and Au_1.1_/Cu_2_O NCs. The two peaks at 2039 cm^−1^ and 2090 cm^−1^ overlap for the Au_2.7_/Cu_2_O NCs, while the former band decreases in intensity until −0.4 V.

At more cathodic potentials, new bands appear at 280 cm^−1^ and 366 cm^−1^, which correspond to the restricted rotation of *CO on Cu (CO_rot_) and Cu–CO stretching (CO_stretch_), respectively. Following our previously established link of their intensity ratio (CO_stretch_/CO_rot_) to the formation of C_2+_ products,^38^ we determined CO_stretch_/CO_rot_ ratios for the Au-decorated Cu_2_O NC catalysts, which are lower than those found previously for the pure Cu_2_O NCs^38^ as well as those for the Ag/Cu_2_O NCs during CO_2_RR.^9^ We also did not detect a potential-dependent correlation between the CO_stretch_/CO_rot_ ratios and the C_2+_ product FE for the Au_*x*_/Cu_2_O NCs as was the case for the bare Cu_2_O NCs or Ag/Cu_2_O NCs, Fig. S23 (ESI†). This result suggests a lower CO coverage on Cu for the Au-decorated Cu_2_O NC catalysts as compared to the pure Cu_2_O NCs^38^ and Ag/Cu_2_O NCs,^9^ and indicates a more complicated mechanism. Thus, the enhanced C_2+_ (liquid) product FE identified for low Au loadings on the Cu_2_O does not appear to stem from differences in the CO coverage on the Cu surface, as it was the case for the Ag-decorated Cu_2_O NCs. Instead, our new findings for the Au–Cu system suggest a more complex mechanism involving the presence of the more easily formed CuAu alloy as compared to the Cu/Ag system. Notably, bands between 500 cm^−1^ and 700 cm^−1^ develop during CO_2_RR, which has not yet been unambiguously assigned and might evolve from Cu/Au–OH species.^41,50^ These bands display a shift towards higher Raman frequencies with increasing Au loading without a specific trend in the intensity, and suggest a decreasing OH binding energy with increasing Au loading.

Fig. 6b shows the Raman spectra for the different Au loadings at −0.6 V and verifies that the C–O stretching bands have similar Raman shifts of ∼2078 cm^−1^. At −1.0 V, the C–O stretching bands of the Au_2.7_/Cu_2_O NC and the Au_1.1_/Cu_2_O NC blue-shift towards 2047 cm^−1^, Fig. 6c. For these high Au loadings we can also identify two additional bands at ∼1900 and 2200 cm^−1^,^51^ which stem from the stretching vibrations of CO on Au sites and can be linked to the significant increase of the FE of CO. For the Au_0.8_/Cu_2_O NC, the C–O stretching band shifts only to 2058 cm^−1^, while no peak shift is observed for Au_0.4_/Cu_2_O NC and the pure Cu_2_O NC with 2088 cm^−1^.^9,41^ Interestingly, the Au_0.4_/Cu_2_O NC shows an additional weak Raman band at ∼2017 cm^−1^, suggesting the presence of multiple CO adsorption sites. The position of the C–O stretching band is directly linked to the average CO binding energy to the surface^50^ and thus, the observed variation in its position during CO_2_RR towards lower Raman shifts reflects a weaker binding of the CO with increasing Au loading, which appears to stabilize for Au loadings higher than 1.1 at%. This effect agrees with the increasing fraction of CuAu alloy with increasing Au loading which exhibits a weaker CO binding energy and can be explained with a downshift of the d-band center from the Fermi level with an increasing Au fraction.^52,53^ It has also been discussed that adjacent OH adsorption to the CO_ads_ sites may influence the C–O stretching band, but we did not detect a link between the C–O band position and the intensity of the Cu/Au–OH band.^54^ Thus, we attribute the variations in the C–O band position to variations in the CO binding primarily induced by CuAu alloy formation, leading to a weaker bound CO on the catalyst surface for the higher Au loadings. For low Au loadings, multiple *e.g.* Cu- and Au-like adsorption sites with clearly different binding energies are present.

Lastly, we have to note that the intensity of the C–O stretching bands at high overpotentials, thus, highest C_2+_ product yield, increases with Au loading compared to the pure Cu_2_O NCs, while the Cu–CO related bands do not vary strongly in intensity. This suggests that a CO-richer surface and/or microenvironment forms during CO_2_RR in the presence of Au-rich regions, while any increase in CO surface coverage would be linked to Au-related sites as the Cu surface coverage does not increase following the CO_stretch_/CO_rot_ analysis. These results suggest a complex mechanism, where the CO coverage on Cu as well as on the Au-containing adjacent regions will play a role, with the optimum surface composition that should be desirable to stabilize under reaction conditions involving Au–Cu alloyed regions in close proximity to Cu regions that would benefit from CO spillover.

Overall, our comprehensive study of the active catalysts state and the catalytic function of Au-decorated Cu_2_O NC suggest that there is a two-stage catalytic role of the Au decoration within the complex mixed phase between the Cu host, CuAu alloy and Au-rich NPs. Clearly, our Au/Cu_2_O catalysts easily form CuAu alloys *in situ* under CO_2_RR conditions, which can be detected within minutes during CO_2_RR and even after CO_2_RR in the near-surface. A higher Au loading leads to stronger alloy formation and results in a weaker average CO binding to the catalyst surface during CO_2_RR. The weaker (average) binding energy agrees with the d-band theory^52^ and enhances CO production.

**Fig. 7:**
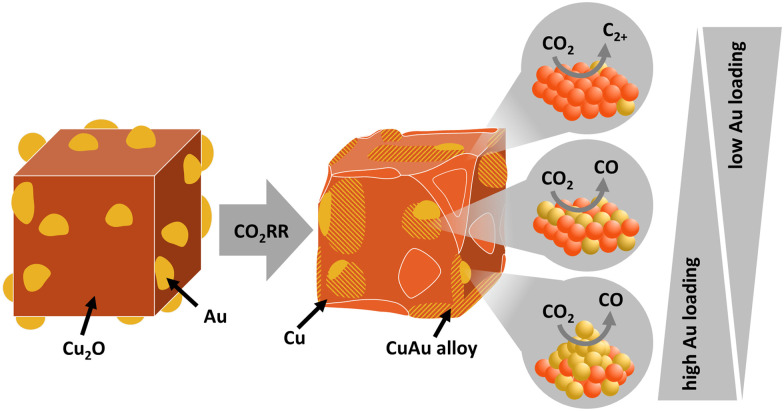
Schematic illustration of the catalyst structure in the as-prepared state and during/after the CO_2_RR, with their corresponding main products. The triangles indicate the amount of Au loading favoring the different Au–Cu atomic structures/redistributions.

This interpretation is in agreement with the current state of knowledge for the formation of C_2+_ products over AuCu,^31,34–36^ which has also been discussed in the literature for CuAg systems,^9,20,40,45,55^ see also Table S12 (ESI†). Homogeneous Cu-rich CuAu alloy generates only a very small fraction of C_2+_ products, while Au-rich alloys produce mostly CO.^31^ Interestingly, the high CO production obtained in the presence of an increasing amount of CO-forming Au and Cu–Au areas has associated the observation of a low CO coverage, as determined from *operando* Raman data. This result suggests a likely weaker binding of CO to the mixed Au–Cu surface obtained for the larger Au loadings, which leads to preferential direct CO desorption *versus* subsequent protonation. Thus, the Au–Cu alloy itself is not considered to improve the CO_2_RR catalytic function for C_2+_ formation, since this occurs on Cu–Cu sites. While CO may be produced in the Au–Cu and Au regions of our catalysts, the actual CO dimerization occurs on the Cu surface in a sequential fashion and an optimal ratio between all regions (Au, the CuAu interface and the Cu–Cu surface) is therefore crucial. The higher fraction of near-surface AuCu alloy likely decreases the density of Cu–Cu sites which are better for C_2+_ product formation and, thus, the formed CO cannot be utilized for dimerization at high Au loadings. Therefore, as depicted in Fig. 7, the beneficial effect of Au with respect to the formation of CO is concurrent with the detrimental effect of the Au–Cu alloy in the subsequent C–C coupling, for which Cu–Cu sites are sacrificed. These results are in agreement with literature indicating a lower formation energy for Au as skin-layer, which would increase the surface area of Au compared to Cu.^56^

Nevertheless, the Au-decorated Cu_2_O NCs did not improve the ethanol performance as much with respect to prior literature reports.^35,36^ However, comparing CuAu and CuAg bimetallic systems for improved C_2+_ selectivity to pure Cu, we observe substantial differences between both systems in the onset potential of CO, the CO production rate as well as their ability to form alloyed structures. Table S12 (ESI†) displays the overall better performances towards C_2+_ products of CuAg systems, which are mainly attributed to CO spillover and/or to a good Ag/Cu miscibility without pronounced alloy formation.^9,20^ In contrast, CuAu systems, with its pronounced alloy formation during reaction conditions, may benefit from synergistic effects between the electronic structure and the morphology of the catalytic system.^33^

Unlike other studies in literature, we present here an Au-loading dependent study and found optimal Au loadings <1 at% for improving the C_2+_ products. Only highly Au-diluted CuAu alloys lead to an enhanced C_2+_ FE. Remarkably, the specific activity for C_2+_ (liquid) product formation increased by up to 400%. In this optimal case, a significant fraction of the Cu domains remains unalloyed and the catalysts exhibit a stronger average or even improved CO binding sites during CO_2_RR. The latter ones can act as active sites for CO dimerization, while the minority CuAu alloyed regions still reduce CO_2_ to CO and the tandem catalytic function boosts the intrinsic C_2+_ formation significantly. Our findings suggest that low Au loadings, *i.e.* decoration on the atomic level, can be a promising approach to further enhance the C_2+_ product formation. Here, we see a striking similarity to our findings on Ag-decorated Cu_2_O NCs, which form disperse Ag sites on the Cu surface even at higher Ag loadings.^9^

Our findings strengthen the hypothesis that (i) optimal amounts of additionally formed CO are beneficial for the formation of C_2+_ liquid products on Cu, (ii) CuAu alloys, formed under CO_2_RR operation mainly contribute to the CO formation but not to C_2_ product formation and (iii) ethylene formation is maintained without addition of Au. We therefore conclude that the CuAu systems are worth exploring further for C_2+_ product promotion, in particular liquid products. When low amounts of Au are available in the vicinity of Cu–Cu sites, the indispensable alloy formation during CO_2_RR is low compared to the pure Cu regions. Sufficiently low amounts of Au allow improved C_2+_ selectivity with CuAu bimetallic systems.

## Conclusion

In summary, we demonstrated that highly-diluted Au-decorated Cu_2_O NCs yield notably enhanced C_2+_ production in the presence of a CO-rich environment around copper, which results from the highly CO producing Au NPs, and due to significant restructuring towards CuAu alloy formation. Under CO_2_RR relevant conditions, Cu_2_O NCs with large amounts of Au NPs demonstrate significant restructuring and redistribution by forming CuAu alloyed frames with increased Au-to-Cu ratios on their surface, which appears to stabilize their initial cubic shape. High loadings of Au on Cu_2_O NCs produce predominantly CO, while a small Au loading leads to an enhancement of C_2+_ products. This is assigned to the favourable coexistence in the latter samples of small superficial areas covered with CO-producing CuAu alloy and Au NPs and large areas in their vicinity consisting of pure Cu sites which make the C_2+_ products.

O*perando* XAS enabled following the alloy formation and observing structural changes between Cu and Au that occur within one hour, which led to a variation of the selectivity trends, in agreement to the long-term reactivity measurements. Increasing Au loadings result in a proportional increase of CO, while low Au loadings lead to a notable increase (4-fold) in C_2+_ liquid products such as ethanol, acetaldehyde, 1-propanol, allylalcohol and propionaldehyde.

With *operando* Raman spectroscopy, we could link the catalytic function, in particular the strong CO formation, to a weaker average CO binding to the catalyst surface in a CO-richer microenvironment and/or higher coverage. The preferred C_2+_ product formation is linked to minority CuAu alloy species being formed in close proximity with the stronger CO binding Cu regions. In contrast to our findings for pure Cu_2_O NCs, we did not detect a clear correlation between the Cu surface coverage with CO and the C_2+_ product formation. Linking to the Ag–Cu_2_O system, we emphasize the importance of dispersed CO-producing sites on a Cu host in order to facilitate the CO_2_RR faradaic efficiency. Thus, it is essential to achieve an in-depth understanding of the atomic scale interaction of Au clusters or single sites with oxide-derived Cu using model systems under CO_2_RR to verify its importance over the CO spill-over mechanism. Understanding the selectivity dependencies on the restructuring of a Cu–Au system, which undergoes continuous transformation under CO_2_RR relevant conditions, provides opportunities for a rational design of highly active and selective bimetallic catalysts. Thus, our work provides crucial input to enable knowledge driven catalyst design of bimetallic CO_2_RR electrocatalysts and in particular to explore the potential of ultralow Au decoration of Cu nanocatalysts towards facilitating specific product distribution in CO_2_RR.

## Author contributions

C. R. conceptualization, data curation, investigation, writing – original draft. A. H. data curation, investigation, writing – review and editing. D. C. data curation, investigation, writing – review and editing. M. R. data curation, investigation, writing – review and editing. H. S. J. investigation, writing – review and editing. D. K. investigation, writing – review and editing. M. L. L investigation, writing – review and editing. S. K. investigation, writing – review and editing. U. H. investigation, writing – review and editing. E. M. D investigation, writing – review and editing. S. W. C. data curation, writing – review and editing. J. T. data curation, investigation, writing – review and editing. D. T. L. A. data curation, writing – review and editing. A. B. conceptualization, investigation, writing – original draft. B. R. C. conceptualization, funding acquisition, writing – review and editing.

## Conflicts of interest

There are no conflicts to declare.

## Supplementary Material

EY-002-D3EY00162H-s001
