# Supplementary material for: Operando insights into correlating CO coverage and Cu–Au alloying with the selectivity of Au NP-decorated Cu2O nanocubes during the electrocatalytic CO2 reduction
Source: EES Catal. 2023 Oct 25;2(1):311–23. doi: 10.1039/d3ey00162h (PMC10782806; doi:10.1039/d3ey00162h)
Supplement: EY-002-D3EY00162H-s001 [file EY-002-D3EY00162H-s001.pdf]

Electronic Supplementary Material (ESI) for EES Catalysis.
This journal is © The Royal Society of Chemistry 2023

Supporting Information

Operando Insights into correlating CO Coverage and Cu-Au Alloying with the Selectivity of Au NP decorated Cu_2O Nanocubes during the Electrochemical CO_2 Reduction

Clara Rettenmaier[1], Antonia Herzog[1], Daniele Casari[2], Martina Rüscher[1], Hyo Sang Jeon[1], David Kordus[1], Mauricio Lopez Luna[1], Stefanie Kühl[1], Uta Hejral[1], Earl M. Davis[1], See Wee Chee[1], Janis Timoshenko[1], Duncan T.L. Alexander[2], Arno Bergmann[1], Beatriz Roldan Cuenya[1]

[1] Department of Interface Science, Fritz-Haber-Institute of the Max-Planck Society, Faradayweg 4-6, 14195 Berlin, Germany

[2] Electron Spectrometry and Microscopy Laboratory (LSME), Institute of Physics (IPHYS), École Polytechnique Fédérale de Lausanne (EPFL), Lausanne CH-1015, Switzerland

***Corresponding Author:** roldan@fhi-berlin.mpg.de

Experimental Section

1. Synthesis of Cu_2O NC and Au_X/Cu_2O NCs

The synthesis of Cu_2O NC and Au_x/Cu_2O NCs were performed following previous reports.[1-3] 4 ml of 0.1 M $CuSO_4$ (Alfa Aesar, >98%) was dispersed in 366 ml H_2O (18 MΩ) under vigorous stirring at room temperature. 14 ml of 1 M NaOH (Alfa Aesar, >97%) was added to initiate the nucleation process with $Cu(OH)_2$. After 10 s, the metal salt was reduced with 16 ml of 0.25 M L-ascorbic acid (Sigma Aldrich, reagent grade). After 12 min, a defined amount (0.8 ml, 1.6 ml, 3.2 ml, 8 ml) for the nominal loadings 0.4at%, 0.8at%, 1.1at%, 2.7at%) of 10 mM $HAuCl_4$ (Alfa Aesar, 99.99%) was added. After 13 min, the solution was centrifuged and washed two times with a EtOH:H_2O mixture and two times with ethanol. The catalysts were stored in 20 ml ethanol. The resulting Cu and Au concentrations in each solution were determined by ICP-MS.

2. Electrode Preparation

The electrodes for H-type cell measurements were prepared on 2 cm^2 carbon paper (Alfa Aesar, Toray Carbon Paper, GGP-H-60). The stock solutions were drop-casted on each side of the carbon paper to yield a Cu loading of 50 µg on the electrode for each catalyst. The electrodes were dried over night to ensure complete evaporation of ethanol.

3. Electrochemical Characterization (CVs, DLC)

The electrochemical characterization was performed in a H-type cell in CO_2 saturated 0.1 M $KHCO_3$ (Alfa Aesar, 99.7%-100.5%) and in a one compartment cell, equipped with a leak-free Ag/AgCl (3.4M, LF-1, Alvatek) and an Au wire as counter electrode in Ar saturated 0.1 M NaOH. To control the potential, a Biologic SP-300 was used.

The double layer capacitance (DLC) was measured after the CO_2RR measurements in CO_2 saturated 0.1 M $KHCO_3$. The potential was cycled at increasing scan rates of 10, 20, 40, 80 and 160 mV s^{-1} in the non-faradaic region between -0.4 V and -0.25V vs. RHE.

4. Electrocatalytic Characterization

A H-type cell with two compartments separated by an anion exchange membrane (Selemion, AMV, AGC Inc.) was equipped with a leak-free Ag/AgCl reference electrode (LF-1, Alvatek) near the working electrode in the cathodic compartment, while a platinum gauze (MaTecK, 3600 mesh cm^{-2}) served as counter electrode. The cell was filled with a defined amount of 0.1 M $KHCO_3$ (Alfa Aesar, 99.7%) in each compartment, which was prior purified with a cation-exchange resin (Chelex 100 Resin, Bio-Rad) and pre-saturated with CO_2 (4.5 N) for at least 20 min. The CO_2 flow was held constant at 20 ml min^{-1} during CO_2RR. An Autolab (PGSTAT 302N, Metrohm) potentiostat was used for the electrocatalytic characterization. The Ohmic drop was measured with the *i*-interrupt method prior to the electrocatalytic protocol and with electrochemical impedance spectroscopy (EIS) afterwards. Double layer capacitance was also applied after the previously described protocol. A linear sweep voltammogram from the open circuit potential (ca. 0.5V) to the respective reduction potential at 20 mV s^{-1} was applied followed by chronoamperometry for 4000 s. The online gas product detection was started after 60 s of chronoamperometry and repeated every 15 min with a gas chromatograph (GC, Agilent 7890B), which was geared with a thermal conductivity detector (TCD) for H_2 detection and a flame ionization detector (FID) for carbon products. After each electrocatalytic measurement, the liquid products were analyzed with high-performance liquid chromatography (HPLC, Shimadzu Prominence), which was geared with a NUCLEOGEL SUGAR 810 column and a refractive index detector (RID), and with a liquid GC (L-GC, Shimadzu 2010plus), which was geared with a fused silica capillary column and a FID detector. All potentials are referred to the reversible hydrogen electrode (RHE) with the following equation:

$$E_{RHE} = 0.059 * pH_{electrolyte} + E^{0}_{Ag/AgCl}$$

The pH of CO_2 sat. electrolyte is pH 6.4, E^0 of the used Ag/AgCl electrode was 200 mV.
The Faradaic Efficiency of each gas product x (FE_x) was calculated as

$$FE_x = \frac{\dot{v}\, C_x\, z_x\, F}{A\ V_M\, j_{total}} * 100,$$

while liquid products were calculated as

$$FE_x = \frac{V\ \Delta C_x\ z_x\, F}{\Delta Q} * 100$$

with A: geometric area of the electrode (cm^2), C_x: volume fraction of the product x detected by the GC, F: Faradaic constant (C mol^{-1}), j_{total}: total current density during CO_2RR (A cm^{-2}), ΔQ: total charge transfer (C), $\dot{v}$: CO_2 gas flow rate (L s^{-1}), V: Volume of the electrolyte (L), V_M: molar volume.

5. Inductively Coupled Plasma – Mass Spectrometry (ICP-MS)

The catalyst concentration, as well as the atomic compositions of Cu and Au were determined by ICP-MS (Thermo Fisher iCAP RQ). The samples were digested by adding a mixture of acids (1:1:3 H_2SO_4:HNO_3:HCl) into a known amount of the catalyst and heated to 180°C for 30 min using the digestion Microwave Multiwave GO from Anton Paar. Samples from the electrode were digested with the carbon paper, which was discarded afterwards. The stock solutions, samples from the electrode and the electrolyte samples were diluted 3.33, 19 and 4 times in 3% HCl, respectively.

6. Transmission electron microscopy and energy-dispersive X-ray spectroscopy

The acquisition of scanning transmission electron microscopy (STEM) images and energy-dispersive X-ray spectroscopy (EDXS)maps were performed with a FEI Talos F200X microscope equipped with a XFEG field emission gun and operated at an acceleration voltage of 200 kV. STEM images were acquired using a high angle annular dark field (HAADF) detector, while the EDXS data were recorded using the Super-X 4 quadrant silicon drift detector (SDD) system. All data were acquired using Thermo Fisher Scientific Velox software.

For atomic resolution imaging and STEM-EDXS elemental mapping, the measurements were performed on a double aberration-corrected FEI Titan 60-300 TEM, operated at 200 kV under HAADF-STEM conditions. EDXS data were similarly acquired using a Super-X system and Velox software. For the quantified elemental and ratio maps, the EDXS map data were binned and processed using in-house scripts based on the HyperSpy Python library. The quantification was based on k-factors generated with Velox, using the Brown-Powell ionization cross-section model.

For the STEM analyses, the as prepared catalysts were drop-casted directly on a Ni lacey carbon grid, while the catalyst after CO_2RR was removed from the carbon paper by sonicating it for a short time in 200 µl isopropanol. 40 µl of the obtained solution was then drop casted on a Ni lacey carbon grid.

7. X-ray Diffraction

A Bruker AXS D8 Advance diffractometer in Bragg-Brentano geometry was used for X-ray diffraction (XRD) measurements with Cu $K\alpha_{1+2}$ radiation and a position sensitive energy dispersive LynxEye XE-T silicon strip detector. XRD patterns were measured in continuous scanning mode in the range between 20 and 100° 2θ, with an increment of 0.02° and a counting time of 1 s/step.

Operando high-energy XRD experiments were performed at beamline ID31 (ESRF, Grenoble). A home-made three electrode cell was based on the thin film approach with continuous electrolyte used equipped with a leak-free Ag/AgCl reference electrode and a Pt mesh counter electrode. CO_2-saturated 0.1M $KHCO_3$ was continuously flown through the spectroelectrochemical cell. An X-ray energy of 67 keV and the working distance of the Dectris Pilatus CdTe detector was calibrated using a CeO_2 reference material. The 2D diffraction pattern were integrated using the pyFAI software package and Rietveld refinement performed using TOPAS (Bruker-AXS, v6). The sample was deposited on highly-oriented pyrolytic graphite electrodes with a loading of ~0.1 mg/cm². The diffraction pattern were recorded in grazing-incidence configuration with the incidence angle optimized for best sample to substrate signal ratio. Rietveld refinements using the software package TOPAS® (Bruker-AXS) were

performed for analysis considering the instrumental broadening of the lab diffractometer, zero error and a sample displacement.

8. Quasi *in situ* X-ray photoelectron spectroscopy

Quasi *in situ* X-ray photoelectron spectroscopy (XPS) measurements were performed in an ultrahigh vacuum (UHV) chamber, geared with a commercial Phoibos100 analyser (SPECS GmbH, E_{pass} = 15 eV) and a XR50 (SPECS GmbH) X-ray source with an Al anode ($E_{K\alpha}$=1486.7 eV). The spectra were aligned using Cu^0 (932.67 eV) as reference and fitted using a Shirley-type or a linear background subtraction for X-ray photoelectron or Auger electron spectroscopy, respectively. Quasi *in situ* XPS experiments were performed in a one compartment cell, which was directly attached to the UHV chamber. After CO_2RR, the catalyst was washed with Ar-sat. H_2O to remove residual electrolyte and transferred quickly into UHV under Ar atmosphere to avoid exposure to air and the possible subsequent reoxidation. The electrochemical measurements were carried out using a potentiostat (Autolab PGSTAT 302N, Metrohm).

9. *Operando* X-ray absorption fine-structure spectroscopy

Operando X-ray absorption fine-structure spectroscopy (XAFS) measurements were performed at beamlines located at the synchrotron facilities ALBA (CLAESS beamline), SSRL (BL 2-2 beamline) and SOLEIL (SAMBA beamline) as well as the quick X-ray absorption fine structure (QXAFS) beamline (SuperXAS) at SLS synchrotron facility of the Paul Scherrer Institute, respectively. All experiments were conducted in fluorescence mode at the Cu K-edge (8978.9 eV) and Au L_3-edge (11918.7 eV) with corresponding fluorescence detectors (SI). The *operando* measurements were performed in a three-electrode electrochemical cell (see Ref.[4] for the schematics of the cell) matching the conditions of the selectivity studies. A leak-free Ag/AgCl was used as a reference electrode, while an Pt mesh was used as a counter electrode. The samples were prepared by drop casting 0.25 mg and 10 mg of catalyst on 0.5 cm^2 area of carbon paper with a microporous layer (GDE, Sigracet 39b). Cu K-edge and Au L_3-edge data were collected separately for identical fresh samples with different loadings to optimize the absorption edge signal while avoiding self-absorption. The carbon paper with the deposited catalyst served as a working electrode. It was mounted in the electrochemical cell and fixed with Kapton tape, so that the Kapton-covered carbon paper could act as an X-ray window, while the side coated with the catalyst was in contact with the electrolyte. The measurements for both samples were performed *ex-situ* as well as under *operando* conditions. Energy calibration, background subtraction and normalization of the collected X-ray absorption near-edge (XANES) spectra were performed with a set of home-built Mathematica scripts. The Athena software was used to extract the extended x-ray absorption fine structure (EXAFS).[5] The FEFFIT code was used for EXAFS fitting.[6]

For Au L_3 edge quick XAFS (QXAFS) species were tracked every 1 s and every 100 spectra was averaged to improve the signal quality for $Au_{2.7}/Cu_2O$ NC, while for $Au_{0.4}/Cu_2O$ NC and $Au_{0.8}/Cu_2O$ NC, spectra were collected every 12 min and every two spectra were merged.

10. *Operando* surface-enhanced Raman spectroscopy

For *operando* surface-enhanced Raman spectroscopy (SERS) measurements, a Raman spectrometer (Renishaw, InVia Reflex) equipped with an optical microscope (Leica Microsystems, DM2500M), a motorized stage for sample tracking (Renishaw, MS300 encoded stage), a near-infrared laser (Renishaw, RL785, λ = 785 nm, Pmax = 500 mW), a CCD detector (Renishaw, Centrus) and a water immersion objective (Leica microsystems, 63x, numerical aperture of 0.9), was used. The water immersion objective was covered with a Teflon film (DuPont, 0.013 mm film thickness) to protect it

from the electrolyte. A Si(100) wafer (520.5 cm^{-1}) was used for calibration. The Raman scattering of the Rayleigh-filtered backscattered light was collected in between 100 – 3200 cm^{-1} with a grating of 1200 lines mm^{-1}. Electrochemical measurements were performed following a previous report.[7] The electrochemical cell was equipped with a Pt counter electrode and a leak-free Ag/AgCl reference electrode; the catalyst was drop-casted on a glassy carbon plate, connected from the back side to the circuit. Measurements were performed with a Biologic SP240 potentiostat. CO_2-saturated 0.1 M $KHCO_3$ was used as electrolyte. Spectrum collection was performed with 10 s exposure time. Focus optimization was done by depth scans. Steady-state conditions at the catalyst surface was ensured by waiting at least 10 min before collecting the spectra. Renishaw WiRE 5.2 software was used to baseline-subtract the data with the intelligent spline feature (8th polynomial order) and to remove cosmic rays.

Supplementary Notes

Supplementary Note 1: Electrocatalytic stability tests

To gain insight over the stability of the catalysts, we performed long-term measurements over 20 h at -1.03 V and tracked the changes of the FEs, Figure S11. The initial product distribution is comparable with the data in Figure 2 that were determined after 1 h of CO_2RR. For all catalysts, after the initial activation and stabilization, the gaseous product distributions remain stable over the course of 20h. Nevertheless, a slight decrease in CO formation is observed for $Au_{0.4}/Cu_2O$ NCs and $Au_{0.8}/Cu_2O$ NCs, while CO production remains stable for $Au_{1.1}/Cu_2O$ NCs and increases for $Au_{2.7}/Cu_2O$ NCs, Figure S11c, suggesting a more sluggish catalyst restructuring for low Au loadings. The total liquid products, analyzed after 20 h of CO_2RR, displays a decrease of the total amount of liquids, Figure S11f. The decrease in the total amount of liquid products is suggested to be observed due to the high polarity of the oxygenates and alcohols during the whole measurement time.

Figure S12 additionally shows a comparison of the liquid products after 1h and after 20h, showing *an increase in* product distributions for the Cu_2O NCs, while the product distributions vary upon the addition of Au. In particular, the ethanol and allylalcohol formation is declines by roughly 4 percentage points, while the propanol formation increases slightly. Interestingly the formation of acetaldehyde and propionaldehyde, Figure S12e and f appears mostly during the first hour of CO_2RR.

Supplementary Note 2: Electrochemical characterization

The catalysts were characterized electrochemically by cyclic voltammetry in CO_2 sat. $KHCO_3$ and Ar sat. NaOH (Figure S13) after reduction of the catalyst for 1 h. All catalysts show the characteristic peaks for $Cu^0 \rightarrow Cu^I$ and $Cu^0 \rightarrow Cu^{II}$ oxidation as well as the $Cu^{II} \rightarrow Cu^I$ and $Cu^I \rightarrow Cu^0$ reduction, respectively. Note that the untypical broad reduction peak from $Cu^{II} \rightarrow Cu^I$ overlaps with the thick oxide layer that was produced at high oxidizing potentials. The upper limit was chosen to eventually oxidize Au, which we did not observed in our CVs. In CO_2 sat. $KHCO_3$, no difference in its redox behavior was found compared to Cu foil. In contradiction, a inhibited oxidation process and a positively shifted Cu oxidation towards higher potentials had been suggested.[8, 9] We assign this contradiction to our results to the low amount of alloyed catalysts and to the unordered type of alloy formation.

In Ar sat. NaOH, OH adsorption is observed at 0.63 V vs. RHE for the catalysts and is lacking in the CV for the electropolished Cu foil. Furthermore, the shape of the oxidation peaks shows much broader shapes than the electropolished Cu foil.

Double layer capacitance was measured for the catalysts after 1 h of CO_2RR and compared to the Cu foil to retrieve a roughness factor, Table S6. The catalysts with Au NPs show higher capacitances and

roughness factors than the Cu_2O NCs, which suggests a higher surface area due to the presence of Au. However, metallic Au species and CuAu alloys also contribute to the capacitance, which impedes a clear assignment.

Figures

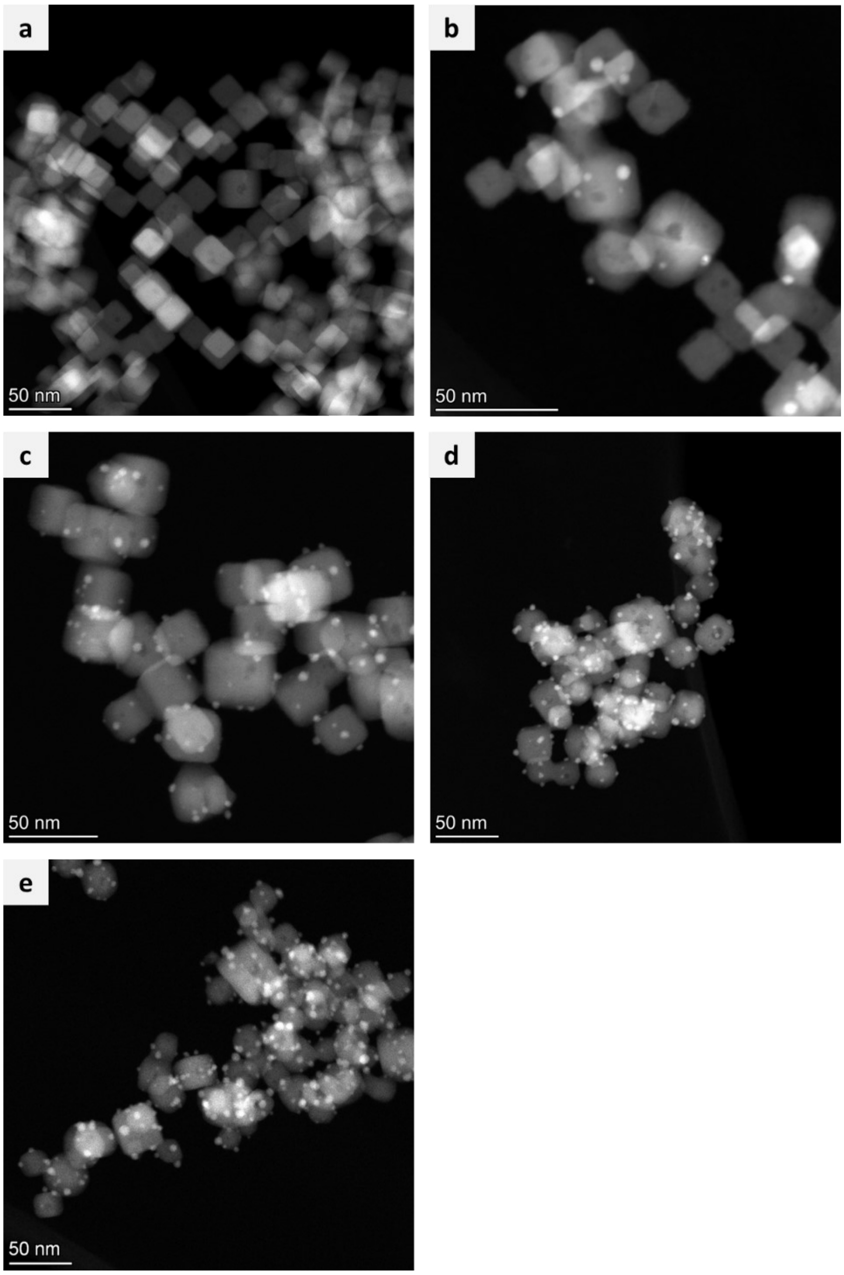

Figure S1. Overview STEM-HAADF micrographs of the catalysts in the as prepared state for a) Cu_2O NC, b) $Au_{0.4}/Cu_2O$ NC, c) $Au_{0.8}/Cu_2O$ NC, d) $Au_{1.1}/Cu_2O$ NC and e) $Au_{2.7}/Cu_2O$ NC.

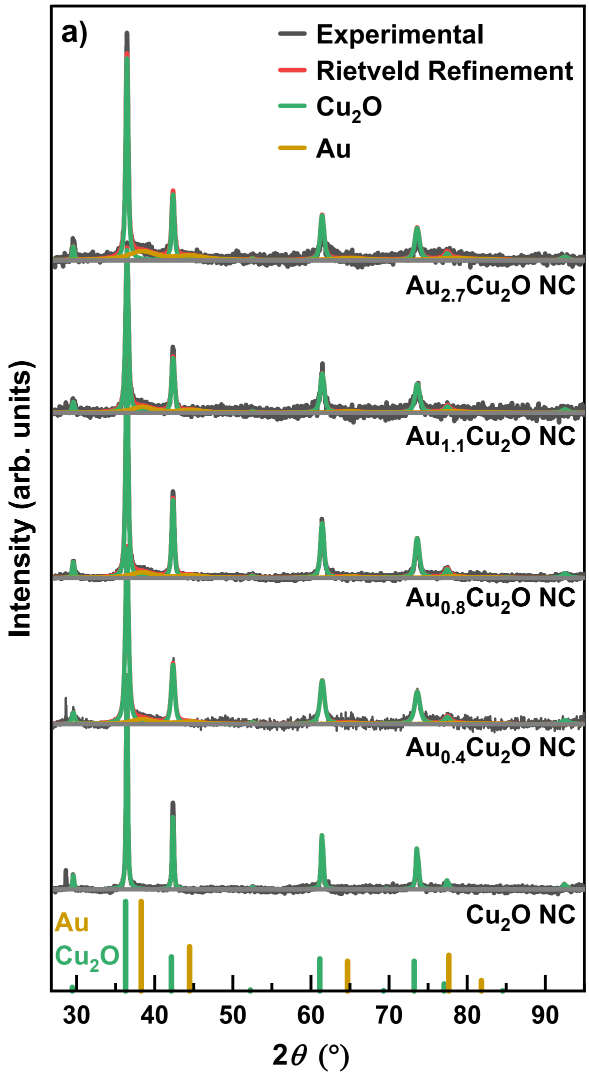

Figure S2 a) *Ex situ* XRD patterns of the Cu_2O NCs and the Au_x/Cu_2O NCs and their corresponding Rietveld refinement fits.

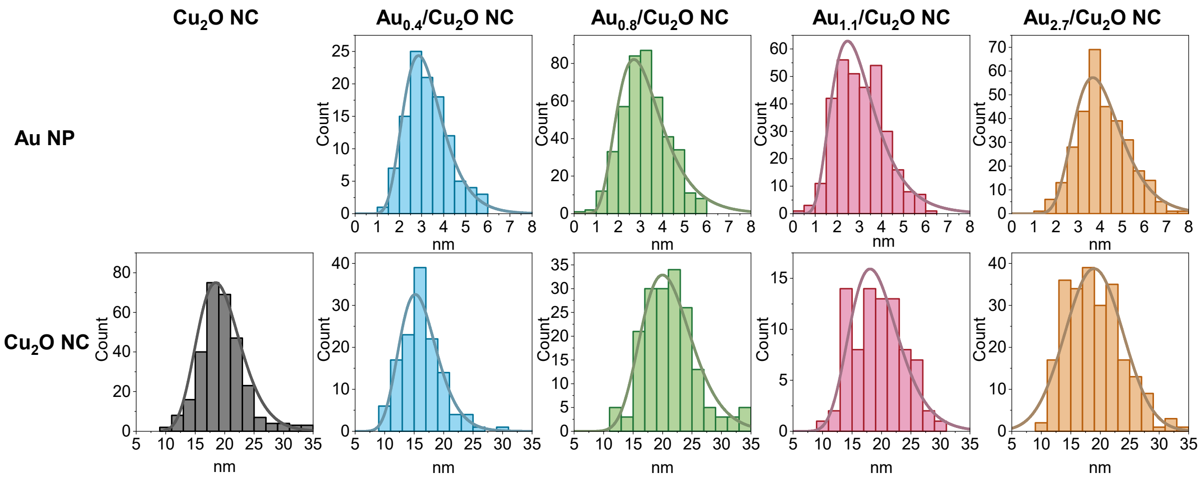

Figure S3. Histograms showing the size distribution of the Cu NC edge lengths and of Au NP diameters for the catalysts in the as prepared state. The size distributions extracted are shown in Table S4.

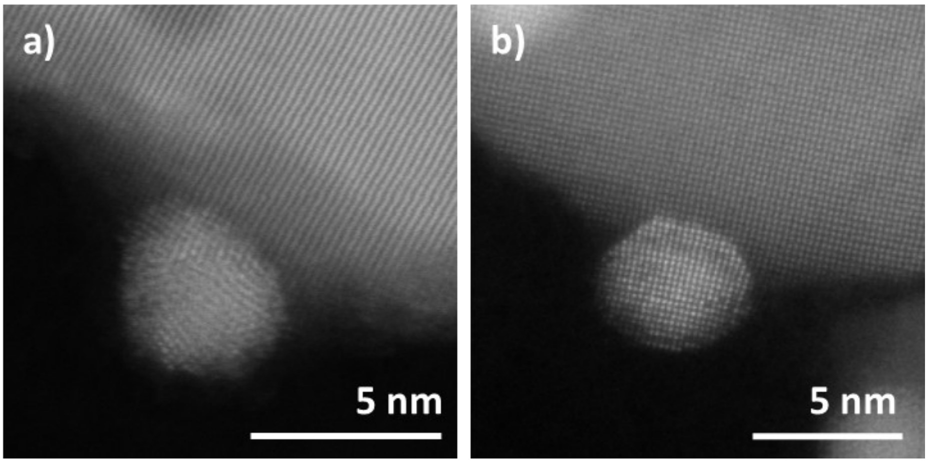

Figure S4. Atomic resolution, aberration-corrected HAADF STEM micrographs of single grain and multigrain Au NPs of $Au_{2.7}/Cu_2O$ NC.

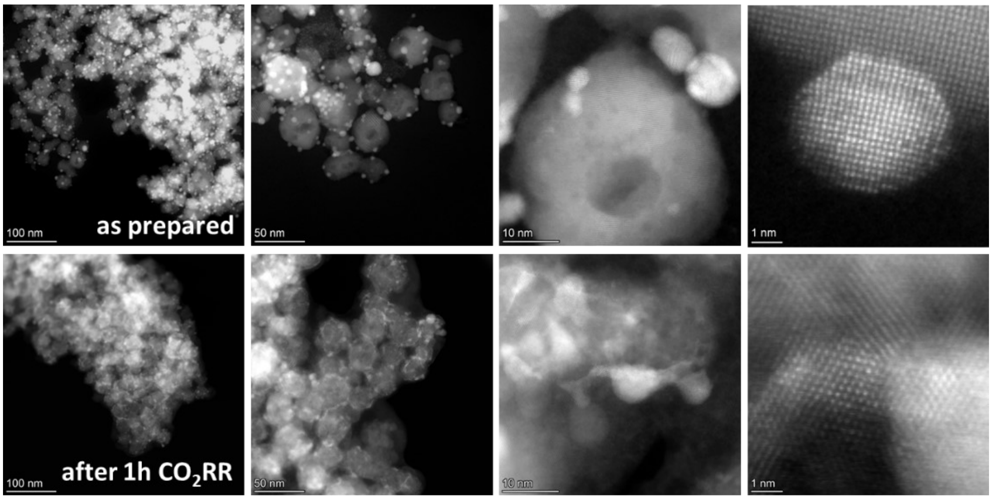

Figure S5. Standard resolution and aberration-corrected atomic resolution STEM-HAADF micrographs of $Au_{2.7}/Cu_2O$ NC a) in the as prepared state and b) after 1 h CO_2RR at -1.0 V vs. RHE.

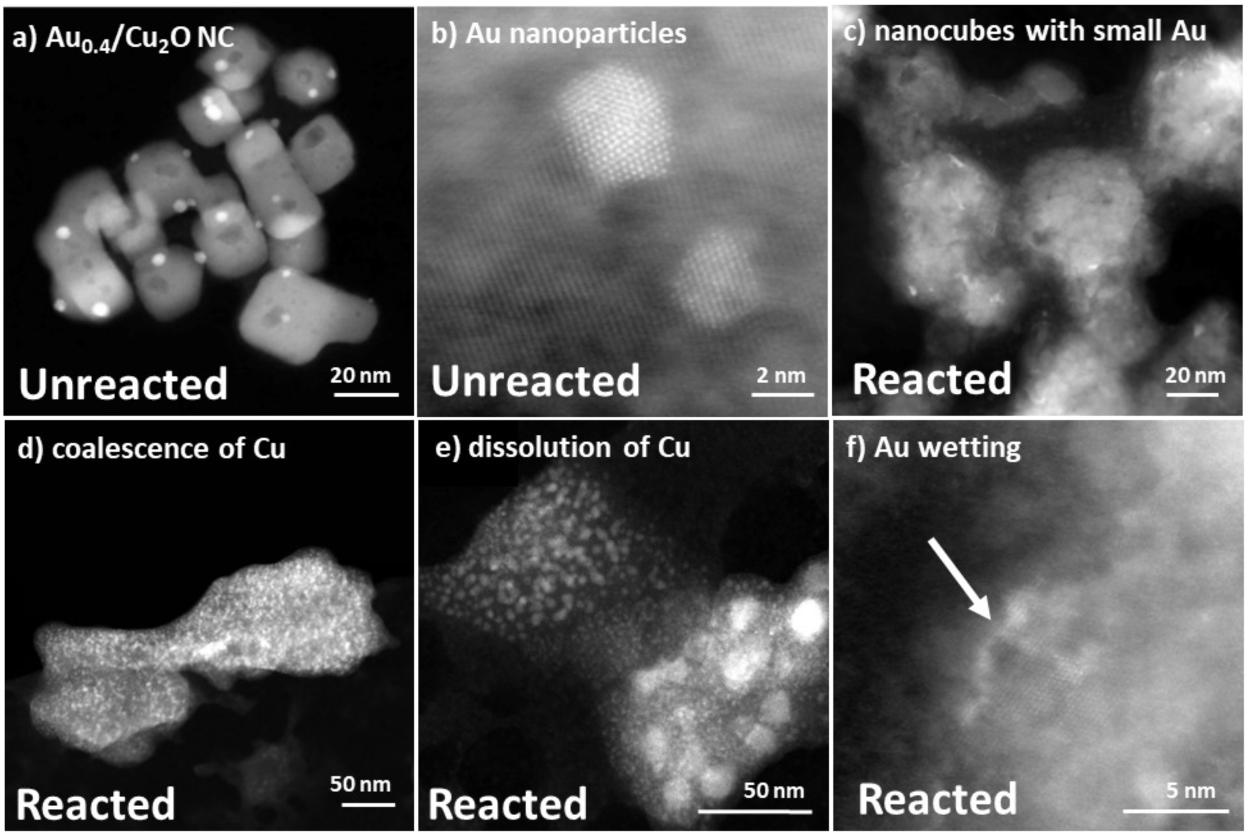

Figure S6. Standard resolution and aberration-corrected atomic resolution STEM-HAADF images of $Au_{0.4}/Cu_2O$ NC a) the unreacted sample, b) unreacted stable Au nanoparticles, c) reacted sample, d) coalescence of Cu and e) dissolved Cu paralleled with f) Au wetting phenomenon.

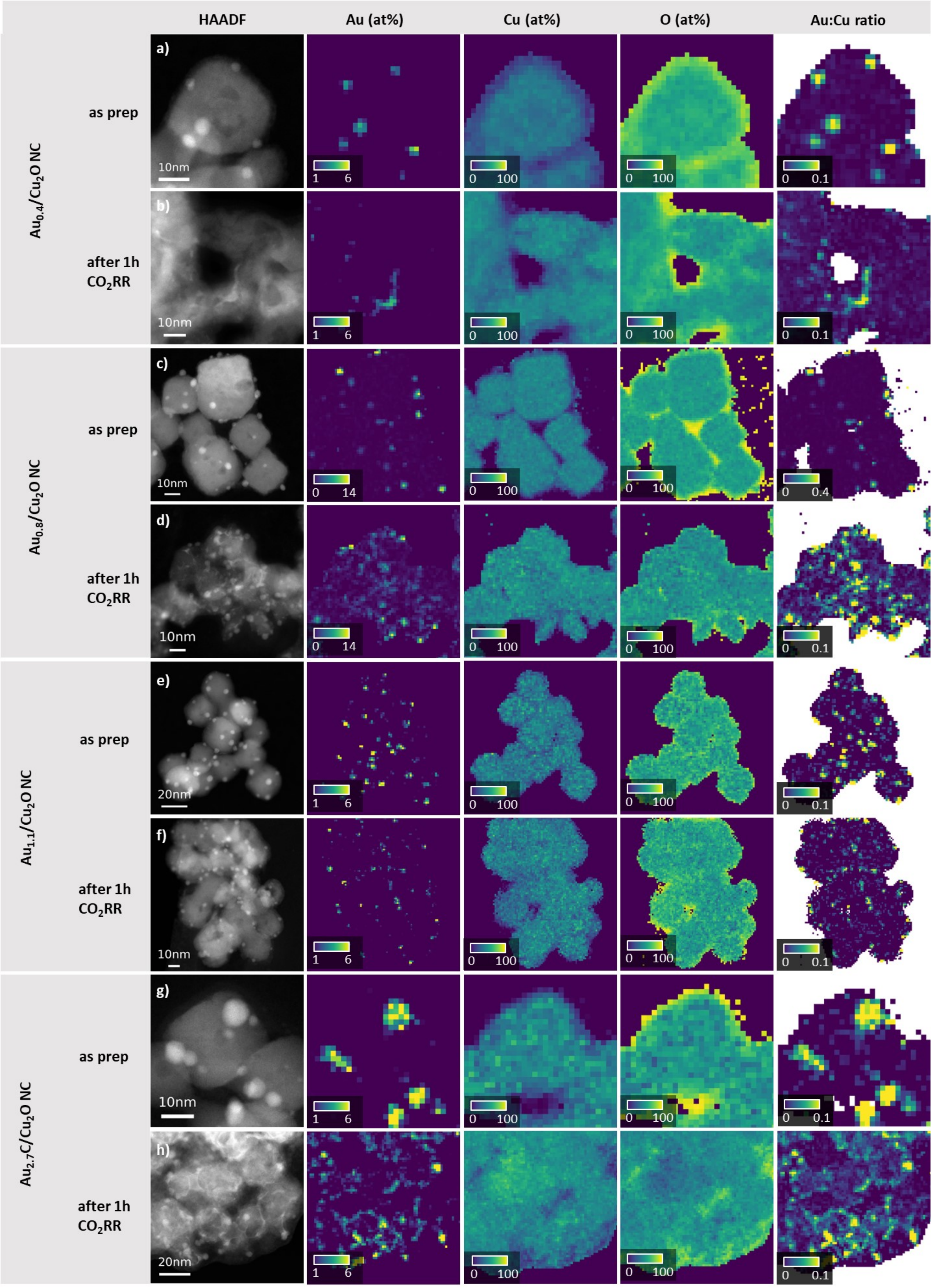

Figure S7. STEM-HAADF micrographs and their corresponding binned and quantified EDXS maps for Au, Cu and O as well as the Au/Cu ratio for all catalysts in the as prepared state and after CO_2RR conditions.

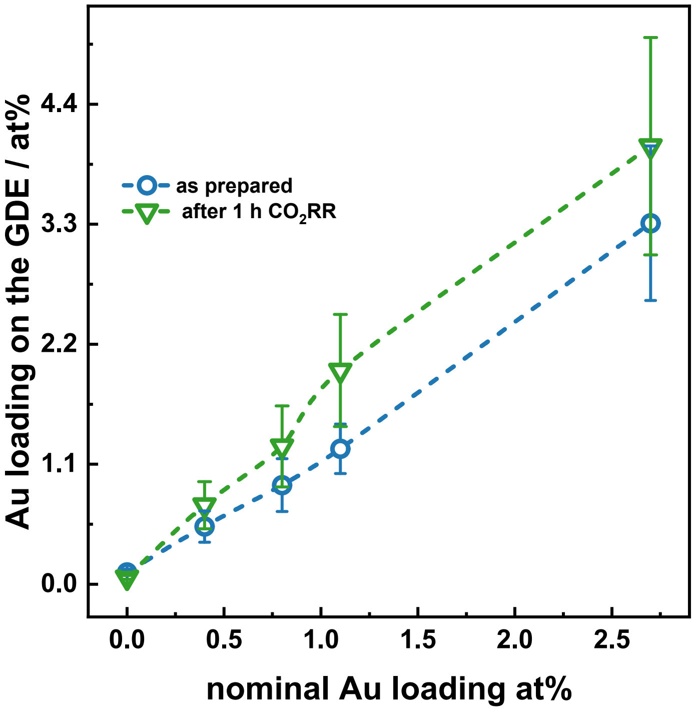

Figure S8. ICP-MS study of the Au loading on the carbon paper support (GDE) in the as prepared state and after 70 min of CO_2RR in at% as a function of the synthesized nominal Au loading in at% in the x-axis. Both axes show percentages of Au compared to the Cu loading.

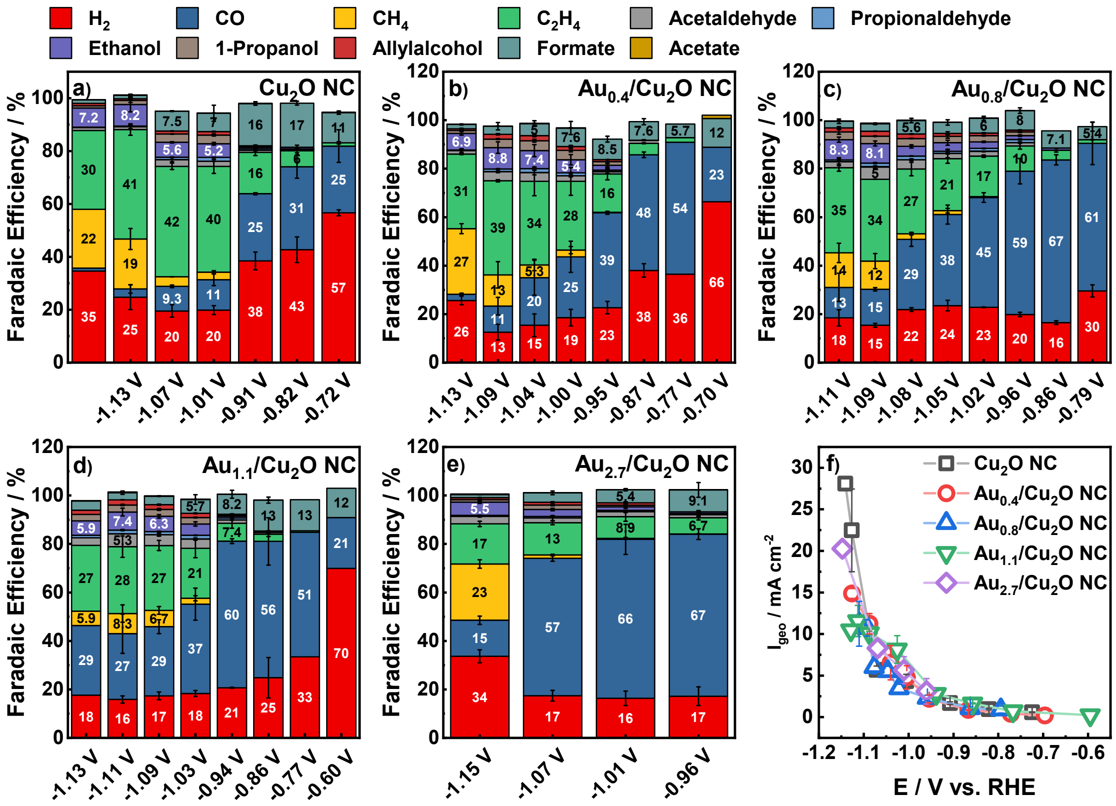

Figure S9. Potential-dependent Faradaic efficiencies for a) Cu_2O NC, b) $Au_{0.4}/Cu_2O$ NC, c) $Au_{0.8}/Cu_2O$ NC, d) $Au_{1.1}/Cu_2O$ NC, e) $Au_{2.7}/Cu_2O$ NC with Nafion. f) Potential-dependent geometric current densities for all catalysts.

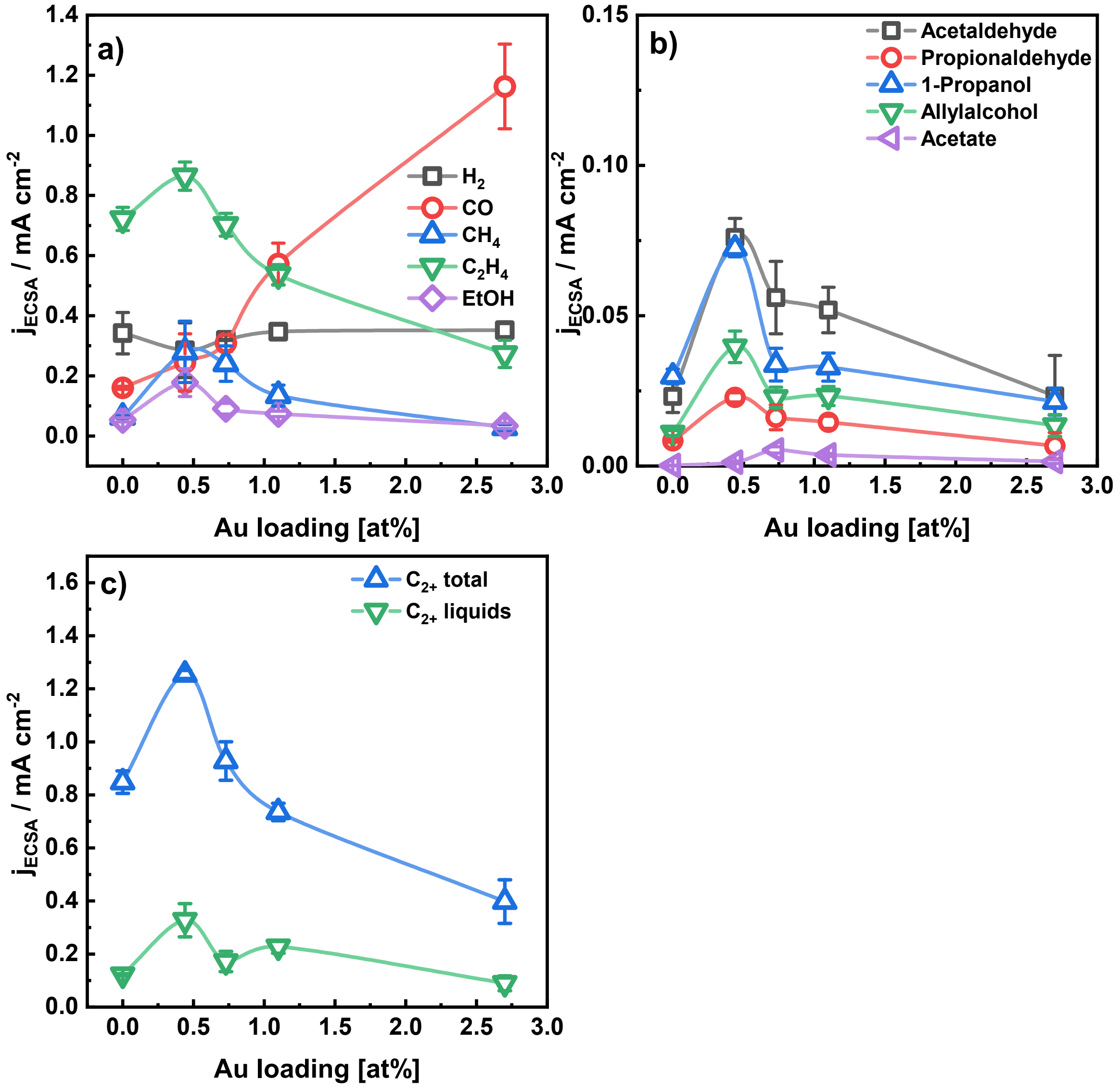

Figure S10. Partial current densities, normalized by the electrochemical surface area from DL capacitance measurements at -1.07V vs. RHE in 0.1 M $KHCO_3$ as a function of the Au NP loading for a) H_2, CO, CH_4, C_2H_4, b) C_{2+} liquids, C_{2+} carbonyls and minor liquid productions, c) the total C_{2+} products and total C_{2+} liquid products

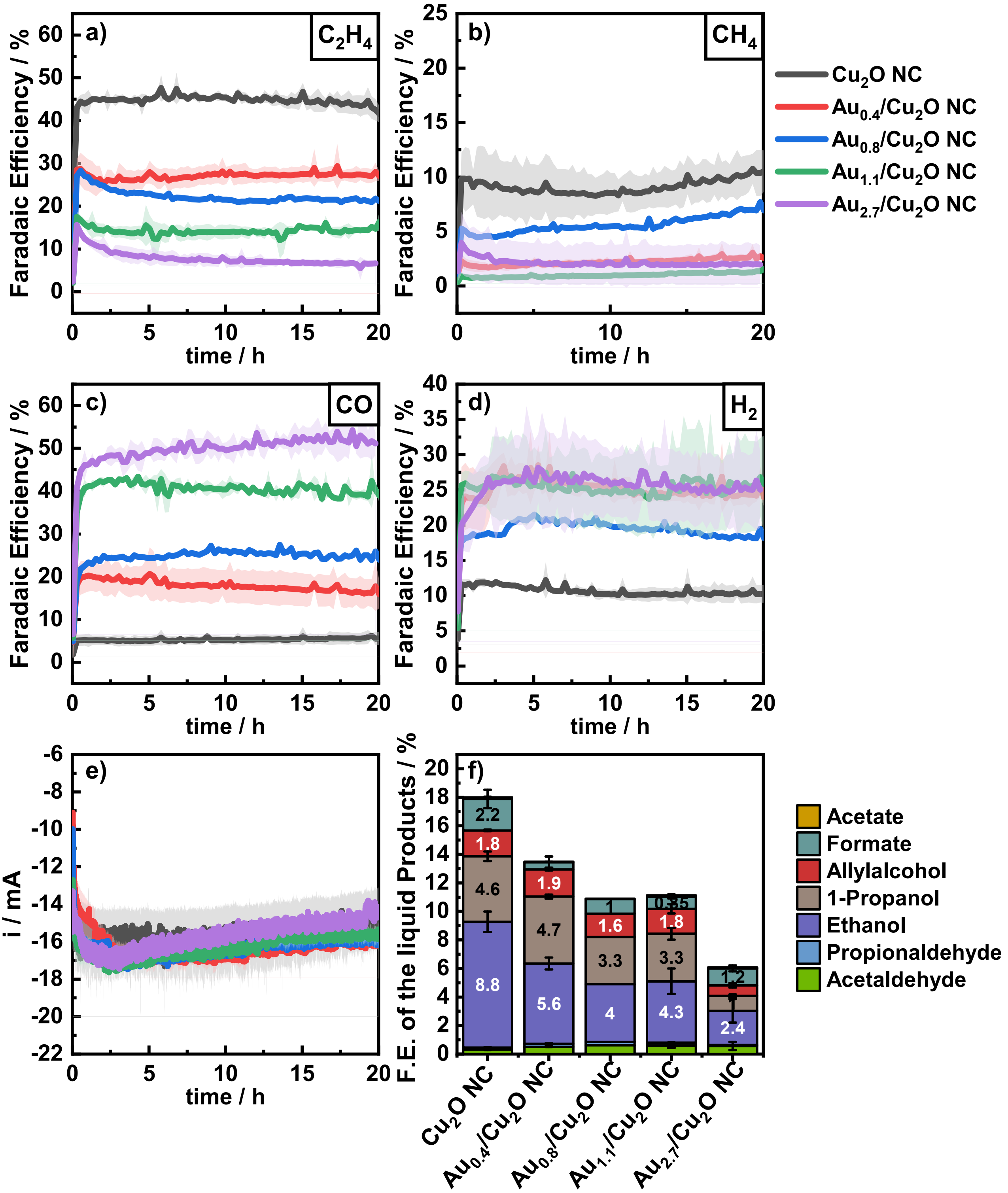

Figure S11. Stability measurements over 20 h at -1.03 V. Faradaic efficiencies of a) C_2H_4, b) CH_4, c) CO and d) H_2; e) current transients and f) liquid products of the catalysts. The gaseous products were analyzed every 15 min using an online GC, the liquid products were analyzed after the 20 h measurement was finished using a liquid GC and a HPLC.

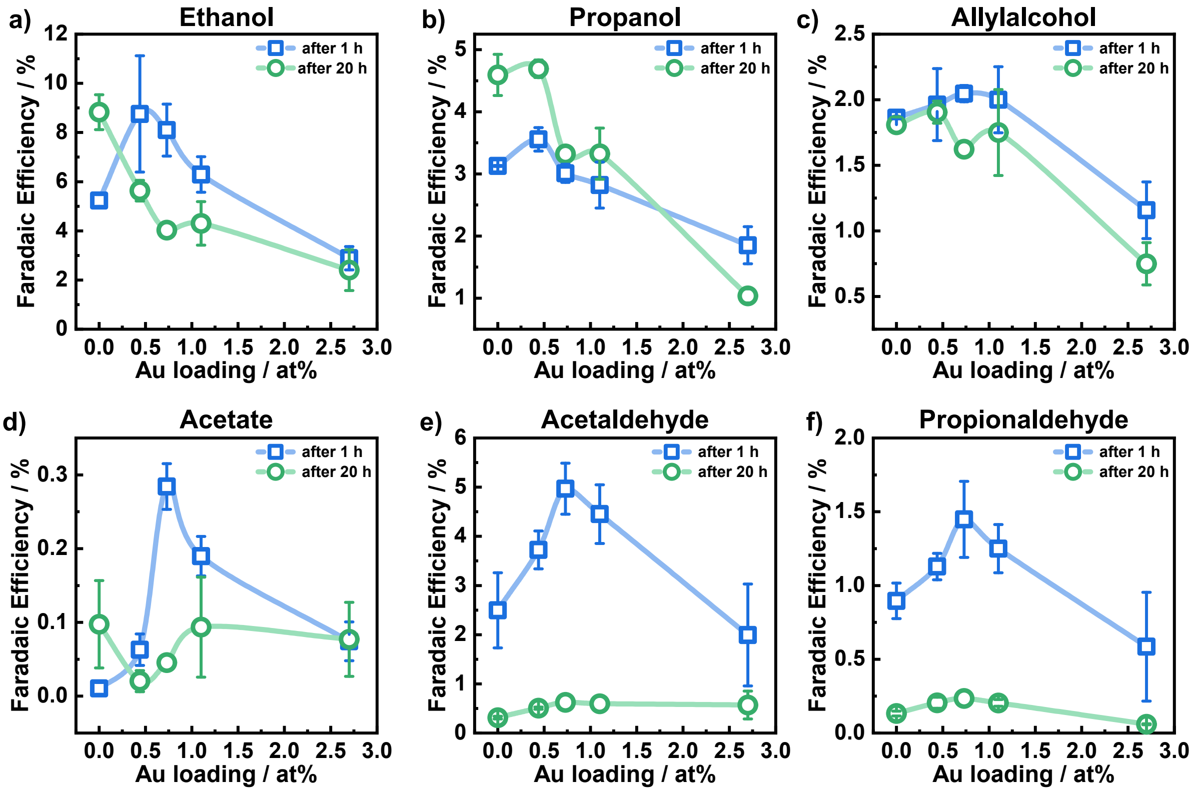

Figure S12: Comparison of the Faradaic Efficiencies of the liquid products a) Ethanol, b) Propanol, c) Allylalcohol, d) Acetate, e) Acetaldehyde and f) Propionaldehyde after 1 h (blue) and after 20 h (green) CO_2RR at -1.05 V vs. RHE.

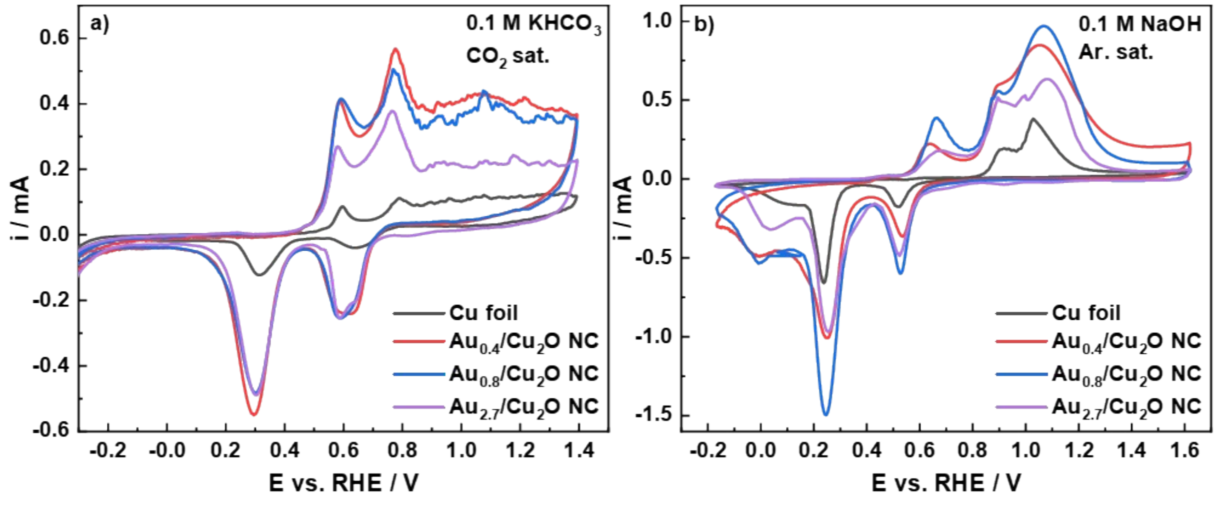

Figure S13. Cyclic voltammograms of the catalysts and Cu foil in CO_2 sat. 0.1 M $KHCO_3$ and in Ar sat. 0.1 M NaOH, scan rate: 20 mV s^{-1}.

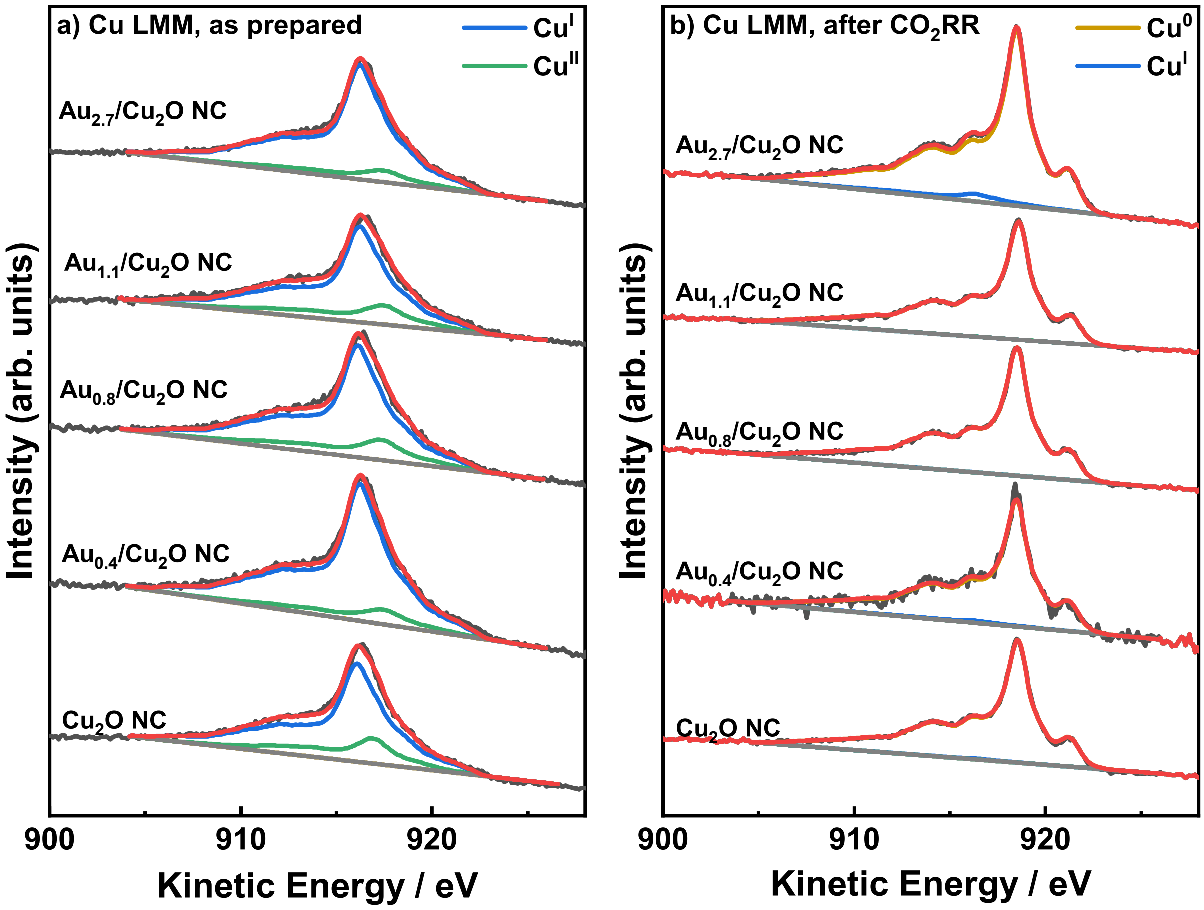

Figure S14. Quasi *in situ* Cu LMM Auger spectra of all catalysts in the a) as prepared state and b) after CO_2RR in 0.1 M $KHCO_3$.

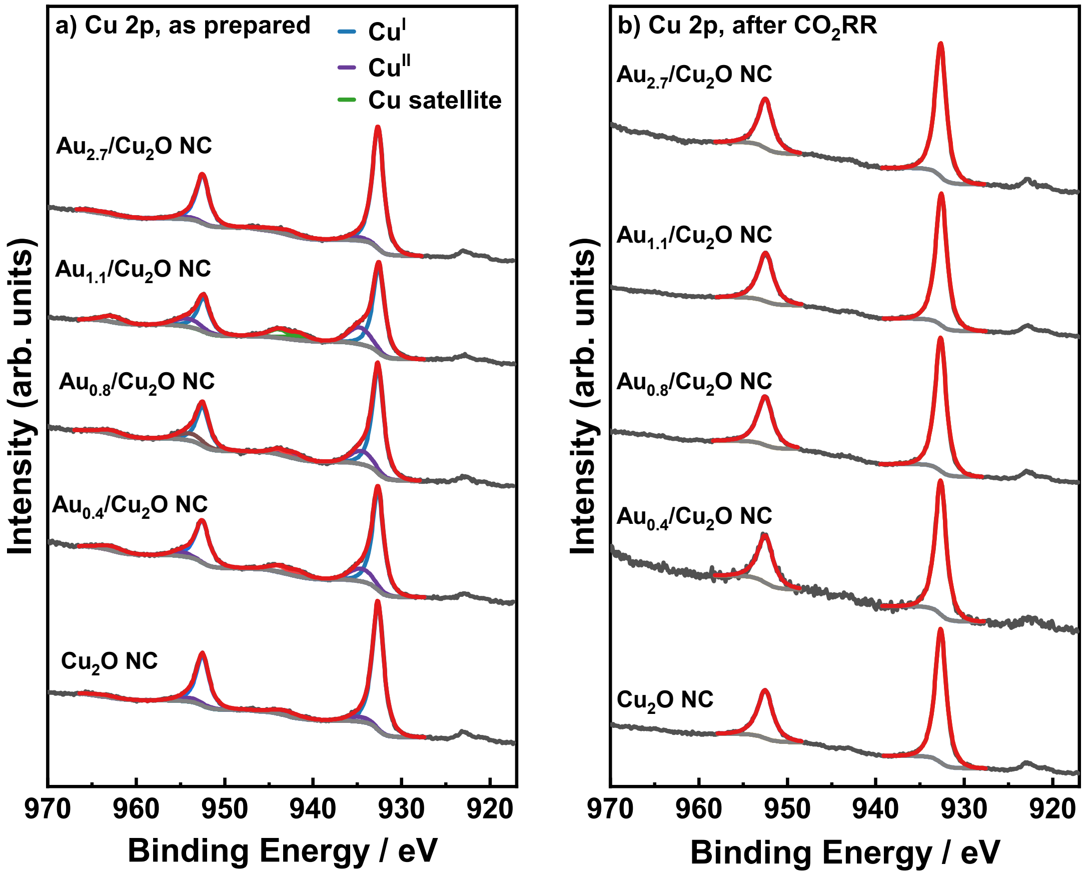

Figure S15. Quasi *in situ* Cu 2p core level regions of all catalysts a) as prepared state and b) after CO_2RR in 0.1 M $KHCO_3$.

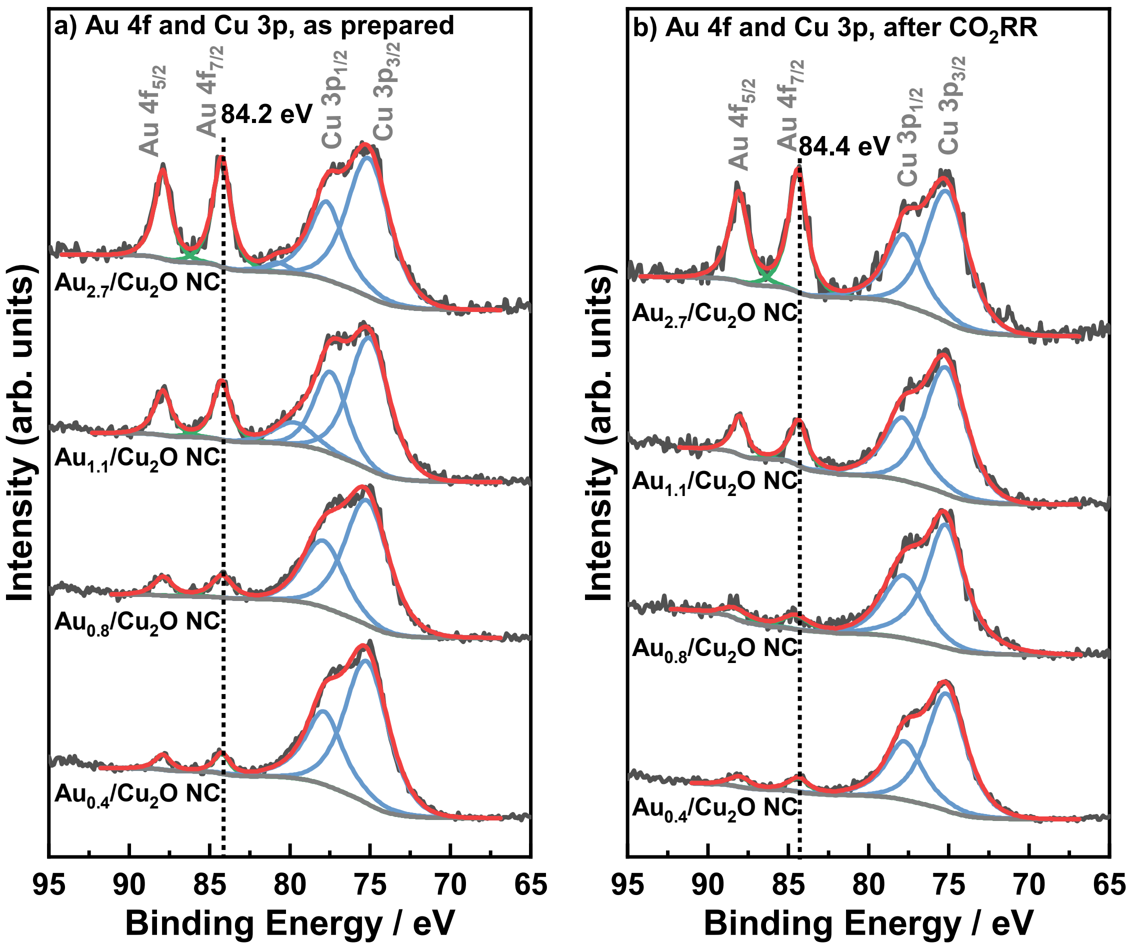

Figure S16. Quasi *in situ* Au 4f and Cu 3p core level regions of all Au-containing catalysts a) in the as prepared state and b) after CO_2RR in 0.1 M $KHCO_3$.

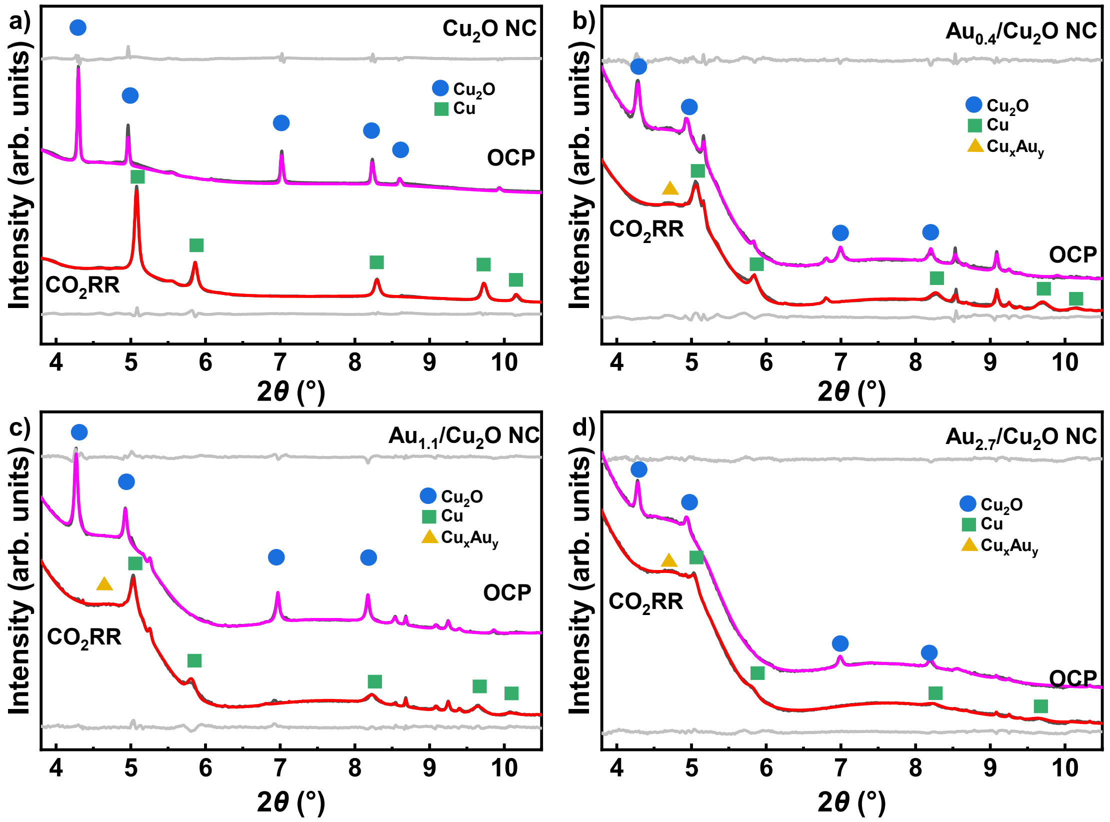

Figure S17. Rietveld refinement of HE-XRD pattern acquired at OCP and during CO_2RR as well as the fitted profiles (pink and red, respectively) of a) Cu_2O NCs, b) $Au_{0.4}/Cu_2O$ NCs, c) $Au_{1.1}/Cu_2O$ NCs, and d) $Cu_{2.7}/Cu_2O$ NC. The difference between experimental data and the fitted profile are shown in grey below and above the pattern recorded at OCP and during CO_2RR, respectively. The X-ray energy was set to 67 keV.

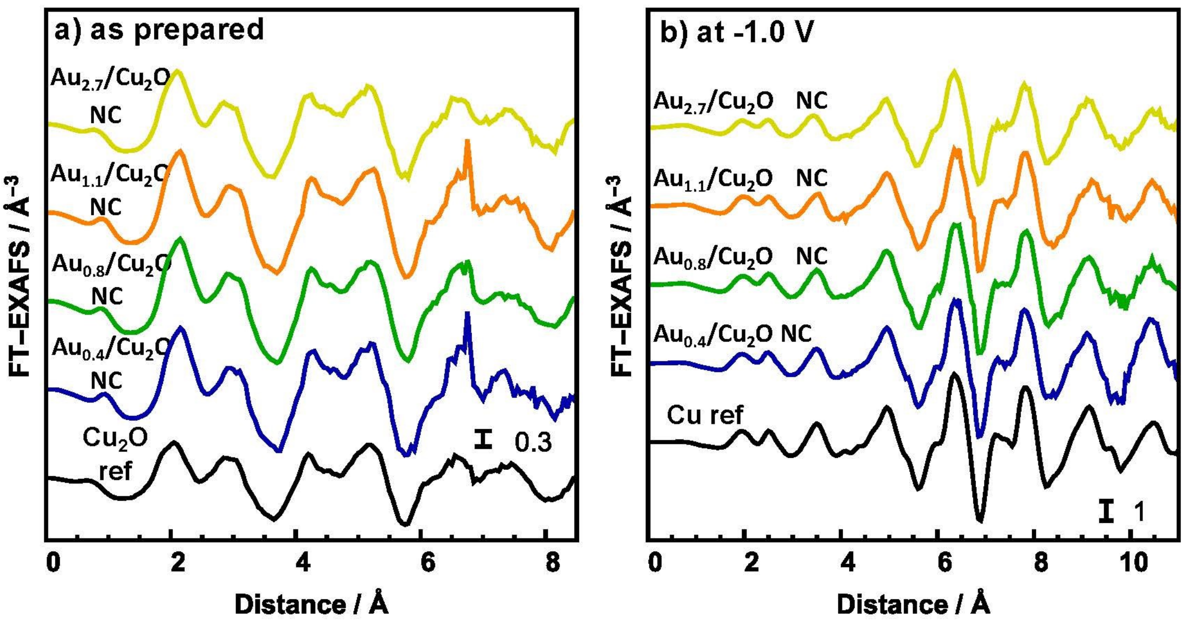

Figure S18. Fourier-filtered Cu K-edge EXAFS spectra in k-space of the Au_x/Cu_2O NC in a) the as-prepared state and b) at -1.0 V. Reference spectra of Cu_2O and Cu are shown for comparison.

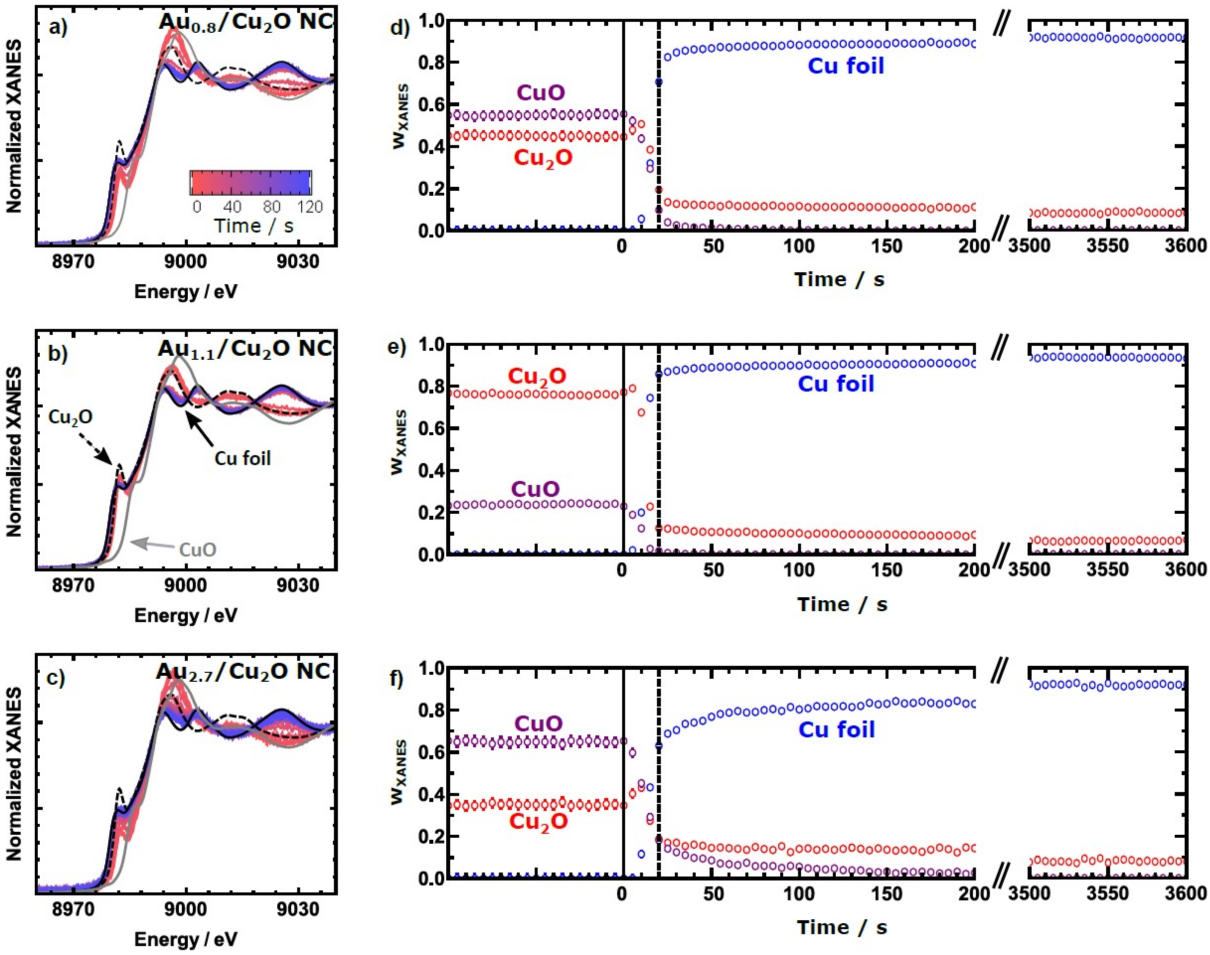

Figure S19. Time dependent Cu K-edge XANES spectra of a) $Au_{0.4}/Cu_2O$ NC, b) $Au_{1.1}/Cu_2O$ NC and c) $Au_{2.7}/Cu_2O$ NC during CO_2RR at -1.0 V vs. RHE and their corresponding time-resolved results of linear combination fitting of the XANES spectra, using spectra for Cu foil, Cu_2O and CuO as references. The samples were measured at OCP before CO_2RR conditions were applied at time 0 s.

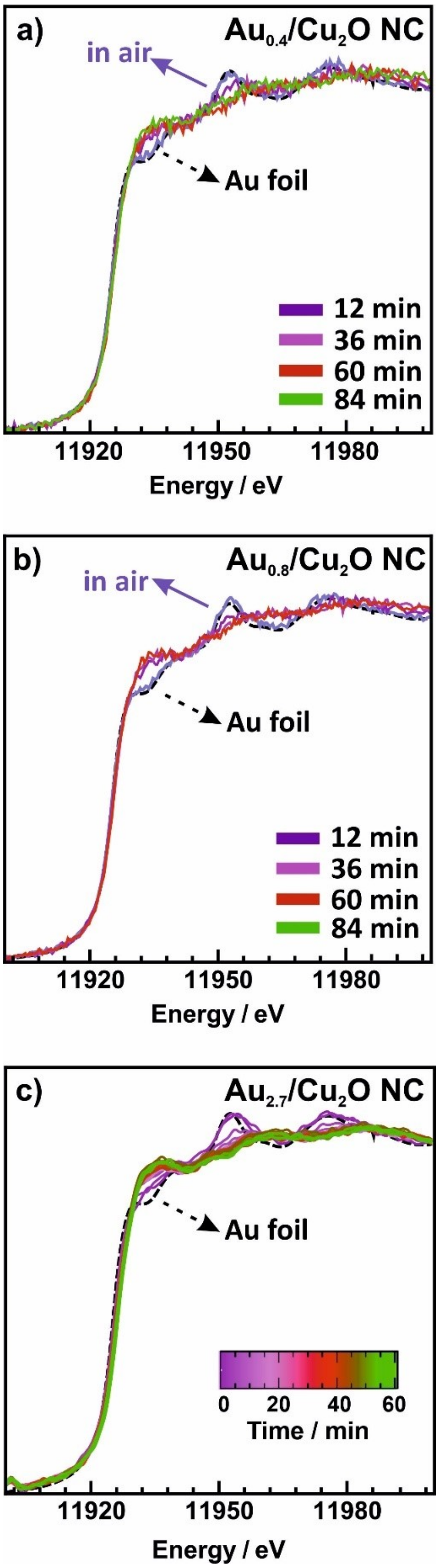

Figure S20. Time dependent Au L_3-edge XANES spectra of a) $Au_{0.4}/Cu_2O$ NC, b) $Au_{0.8}/Cu_2O$ NC and c) $Au_{2.7}/Cu_2O$ NC during CO_2RR at -1.0 V vs. RHE. Reference data from a bulk Au-foil are also shown.

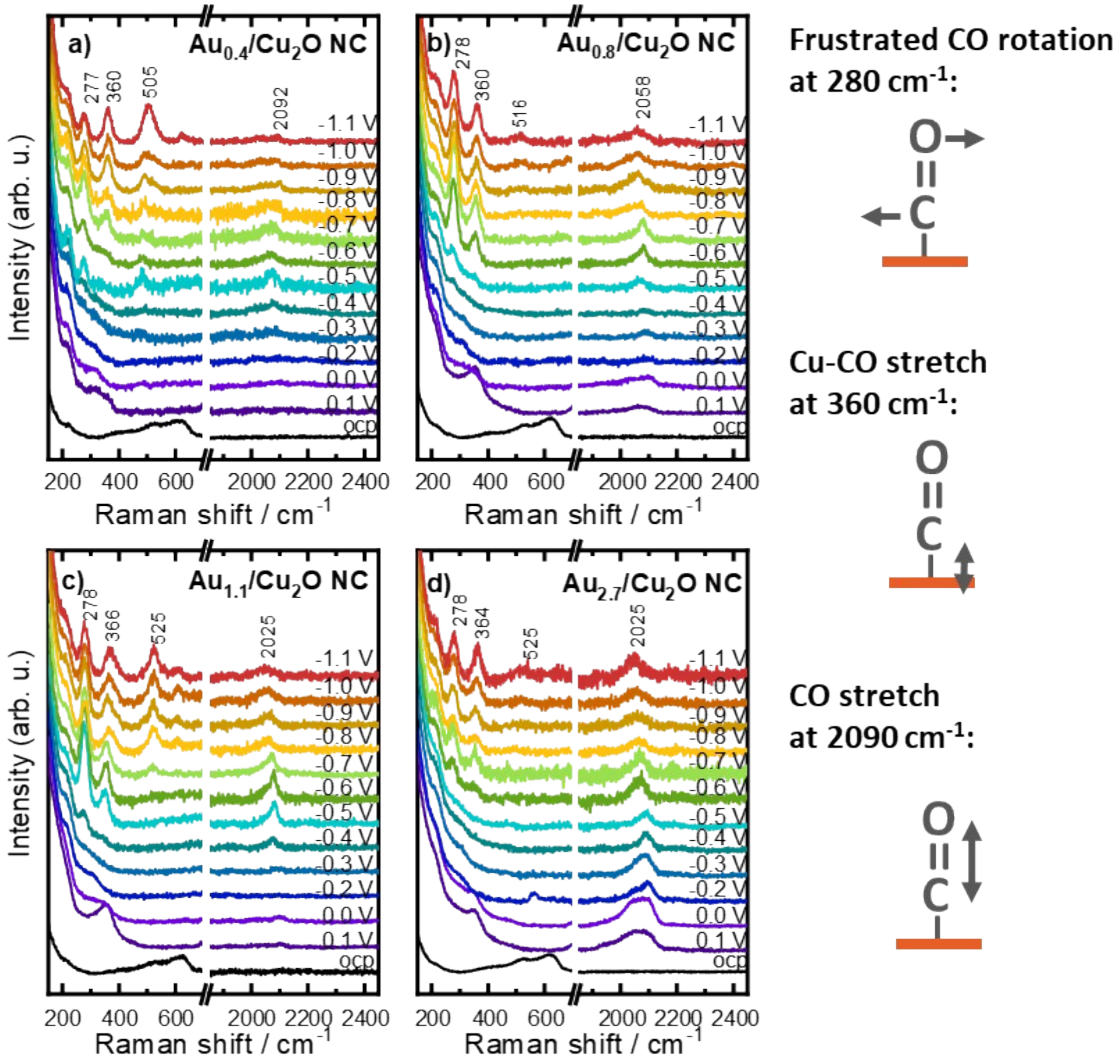

Figure S21. *Operando* surface-enhanced Raman spectra of a) $Au_{0.4}/Cu_2O$ NC, b) $Au_{0.8}/Cu_2O$ NC, c) $Au_{1.1}/Cu_2O$ NC and d) $Au_{2.7}/Cu_2O$ NC in CO_2 sat. 0.1 M $KHCO_3$ with stepped potentials from open circuit potential to -1.1V vs. RHE. Key species are identified at 280 cm^{-1} and 366 cm^{-1}, which correspond to the restricted rotation of *CO on Cu (CO_{rot}) and Cu-CO stretching ($CO_{stretch}$), respectively, as well as CO stretching band at 2090 cm^{-1}.

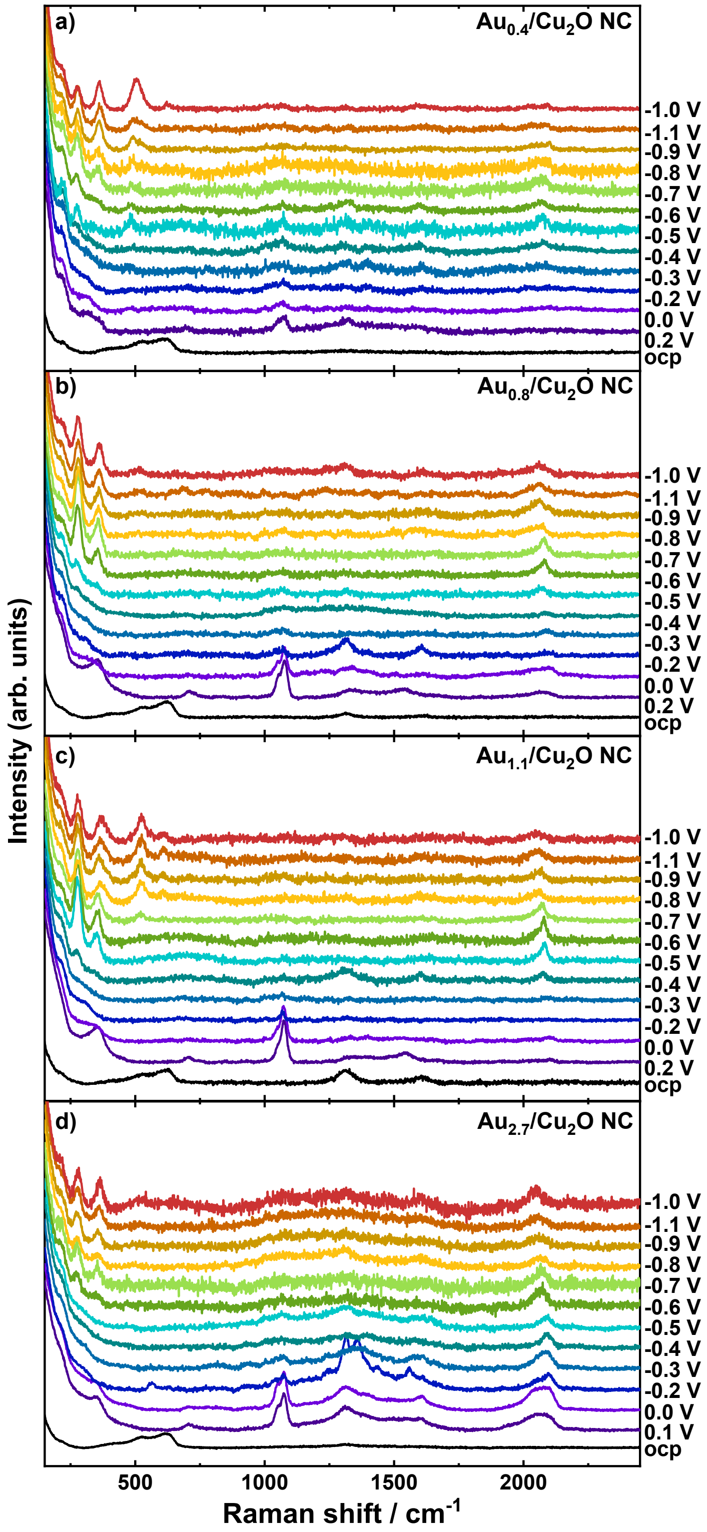

Figure S22. Full Raman spectra of a) $Au_{0.4}/Cu_2O$ NC, b) $Au_{0.8}/Cu_2O$ NC, c) $Au_{1.1}/Cu_2O$ NC, d) $Au_{2.7}/Cu_2O$ NC at increasing applied potentials vs. RHE in CO_2-sat. 0.1 M $KHCO_3$. The region between 1000 cm^{-1} and 1700 cm^{-1} holds vibration peaks of HCO_3^- (1005 cm^{-1}) and CO_3^{2-} (1072 cm^{-1}), and from the glassy carbon support (1313 cm^{-1} and 1600 cm^{-1}).

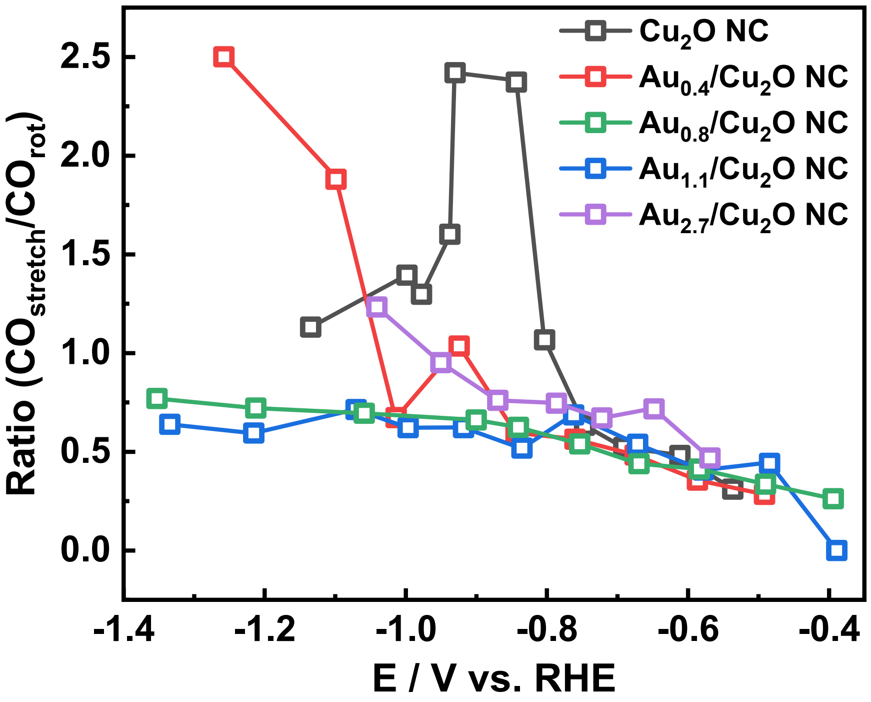

Figure S23. CO$_{stretch}$/CO$_{rot}$ ratios of the respective bands (277 cm^{-1} and 360 cm^{-1}) as a function of applied potential (iR corrected). The results for Cu$_2$O were calculated from Ref [8].

Supplementary Tables

Table S1. Composition of Cu and Au of the presented catalyst dispersions, determined by ICP-MS.

Catalyst	Cu [at%]	Au [at%]
**Cu_2O NC**	100.00 ± 0.01	0.00 ± 0.01
**$Au_{0.4}/Cu_2O$ NC**	99.57 ± 0.00	0.43 ± 0.00
**$Au_{0.8}/Cu_2O$ NC**	99.23 ± 0.01	0.77 ± 0.01
**$Au_{1.1}/Cu_2O$ NC**	98.92 ± 0.02	1.08 ± 0.02
**$Au_{2.7}/Cu_2O$ NC**	97.31 ± 0.04	2.69 ± 0.04

Table S2. Coherence lengths, lattice parameters and atomic fractions of Cu_2O and Au extracted from Rietveld refinement of the *ex situ* XRD pattern of all catalysts in the as prepared state.

Catalyst	Structural coherence lengths /nm		Lattice parameters / Å		Weight Fraction	
	**Cu_2O**	**Au**	**Cu_2O**	**Au**	**Cu_2O**	**Au**
**Cu_2O NC**	20(9)		4.2668(3)		100%	
**$Au_{0.4}/Cu_2O$ NC**	21.3(8)	1.8(2)	4.2670(5)	4.059(4)	93.5(4)%	6.5(4)%
**$Au_{0.8}/Cu_2O$ NC**	35.0(10)	1.78(11)	4.2669(3)	4.059(4)	92.4(2)%	7.6(2)%
**$Au_{1.1}/Cu_2O$ NC**	26.3(16)	1.8(2)	4.2668(7)	4.059(4)	90.5(5)%	9.5(5)%
**$Au_{2.7}/Cu_2O$ NC**	42(4)	1.40(10)	4.2680(7)	4.059(4)	84.7(5)%	15.3(5)%

Table S3. Edge lengths of the Cu NCs and diameters of the Au NP in the as prepared state and after 70 min CO_2RR obtained from STEM-HAADF micrographs.

Edge Length & particle sizes	as prepared		after 70 min CO_2RR	
	Cu / [nm]	Au / nm	Cu / nm	Au / nm
Cu_2O NC	21.8 ± 7.1	--	30.5 ± 4.4	--
$Au_{0.4}/Cu_2O$ NC	16.2 ± 3.4	4.1 ± 1.2	21.4 ± 5.4	3.8 ± 1.5
$Au_{0.8}/Cu_2O$ NC	21.4 ± 4.6	3.6 ± 1.0	25.4 ± 3.8	3.4 ± 1.8
$Au_{1.1}/Cu_2O$ NC	19.5 ± 4.3	3.4 ± 1.0	19.6 ± 4.2	3.8 ± 1.4
$Au_{2.7}/Cu_2O$ NC	18.9 ± 4.9	4.1 ± 1.4	20.2 ± 5.5	3.6 ± 1.6

Table S4. Compositions measured using EDXS comparing the Cu and Au ratios of the catalysts in the as prepared state and after 70 min CO_2RR. Evaluation based on L lines of the elements.

EDX Cu-Au composition	as prepared	after 70 min CO_2RR
	Au [at%]	Au [at%]
Cu_2O NC	0.00	0.00
$Au_{0.4}/Cu_2O$ NC	0.2 ± 0.1	0.5 ± 0.1
$Au_{0.8}/Cu_2O$ NC	0.4 ± 0.1	0.6 ± 0.1
$Au_{1.1}/Cu_2O$ NC	0.6 ± 0.1	0.6 ± 0.1
$Au_{2.7}/Cu_2O$ NC	1.2 ± 0.4	1.4 ± 0.5

Table S5. ICP-MS analysis of the Au:Cu ratio of the catalysts on 2 cm^2 carbon paper electrode in the as-prepared state and after 1 h of CO_2RR at -1.0 V, and of the electrolyte after reaction.

	Au loading in at%	
	as prepared	CO_2RR
Cu_2O NC	0.11 ± 0.03	0.06 ± 0.02
$Au_{0.4}/Cu_2O$ NC	0.5 ± 0.1	0.7 ± 0.2
$Au_{0.8}/Cu_2O$ NC	0.9 ± 0.2	1.3 ± 0.4
$Au_{1.1}/Cu_2O$ NC	1.2 ± 0.2	2.0 ± 0.5
$Au_{2.7}/Cu_2O$ NC	3.3 ± 0.7	4.0 ± 1.0

Table S6. Double Layer Capacitances and corresponding roughness factors (normalized to Cu foil) of the catalysts with Nafion and of the reference Cu and Au foils measured after 1 h CO_2RR at -1.0V vs. RHE.

Sample	Capacitance / µFcm^{-2}	Roughness factor (RF)
**Cu Foil**	29.3	1
**Cu_2O NC**	101.3	3.45
**$Au_{0.4}/Cu_2O$ NC**	181.66	6.2
**$Au_{0.8}/Cu_2O$ NC**	161.0 ± 3.3	5.5
**$Au_{1.1}/Cu_2O$ NC**	150.3	5.1
**$Au_{2.7}/Cu_2O$ NC**	122.7 ± 6.65	4.2

Table S7. XPS composition between Cu, Cu_2O and CuO obtained by linear combination fitting of the Cu LMM spectra in the as prepared state and after 1 .

**XPS composition**	**as prepared**		**after 70 min CO_2RR**	
	**Cu_2O [%]**	**CuO [%]**	**Cu [%]**	**Cu_2O [%]**
**Cu_2O NC**	80	20	99.5	0.5
**$Au_{0.4}/Cu_2O$ NC**	89	11	98	2
**$Au_{0.8}/Cu_2O$ NC**	83	17	100	0
**$Au_{1.1}/Cu_2O$ NC**	82	18	100	0
**$Au_{2.7}/Cu_2O$ NC**	89	11	96	4

Table S8. XPS composition of Cu (all chemical states) and Au in the as prepared state and after 1 h under CO_2RR conditions comparing Cu 3p with Au 4f peaks

**XPS composition**	**as prepared**		**after 70 min CO_2RR**	
	**Cu [%]**	**Au [%]**	**Cu [%]**	**Au [%]**
**Cu_2O NC**	100	--	100	--
**$Au_{0.4}/Cu_2O$ NC**	99.07	0.93	98.25	1.75
**$Au_{0.8}/Cu_2O$ NC**	97.65	2.35	97.34	2.66
**$Au_{1.1}/Cu_2O$ NC**	97.27	2.73	96.12	3.88
**$Au_{2.7}/Cu_2O$ NC**	93.71	6.29	91.46	8.54

Table S9. Coherence lengths, lattice parameters and atomic fractions of Cu_2O and Au extracted from Rietveld refinement of the *operando* XRD pattern of all catalysts in OCP and under CO_2RR conditions.

**Catalyst**	**Structural coherence lengths /nm**		**Lattice parameters / Å**		**Weight Fraction / %**	
**OCP**	**Cu_2O**	**Au**	**Cu_2O**	**Au**	**Cu_2O**	**Au**
**Cu_2O NC**	29(2)	-	4.2819(2)	-	100	-
**$Au_{0.4}/Cu_2O$ NC**	9.2(4)	3(5)	4.2927(7)	3.980(9)	99.0(14)	1.0(14)
**$Au_{1.1}/Cu_2O$ NC**	12.4(2)	1.4(3)	4.3088(2)	4.060(2)	96.1(6)	3.9(6)
**$Au_{2.7}/Cu_2O$ NC**	17.3(13)	3.1(1)	4.2894(9)	4.052(9)	90.0(12	10.0(1)

**Catalyst during**	**Structural coherence lengths /nm**			**Lattice parameters / Å**			**Weight Fraction / %**		
**CO_2RR**	**Cu_2O**	**Cu**	**Au_{1-x}-Cu_x**	**Cu_2O**	**Cu**	**Au_{1-x}-Cu_x**	**Cu_2O**	**Cu**	**Au_{1-x}-Cu_x**
**Cu_2O NC**	-	12.0(2)	-	-	3.6258(1)	-	-	100	-
**$Au_{0.4}/Cu_2O$ NC**	-	6.6(2)	-	4.212(2)	3.631(2)	4(13)	1(4)	99(2)	0(1)
**$Au_{1.1}/Cu_2O$ NC**	-	12.5(2)	0.3(8)	4.2168(3)	3.6501(5)	4.0(18)	0.9(3)	96(6)	3(6)
**$Au_{2.7}/Cu_2O$ NC**	-	7.0(7)	5(5)	4.285(7)	3.644(2)	3.88(2)	1(2)	98(6)	9(5)

Table S10. Structural parameters (coordination number N, interatomic distances R, disorder factors σ[2]) obtained from fitting the Cu K-edge EXAFS data acquired for Au-decorated Cu2O in the as prepared state and during CO_2RR at -1.0 V vs. RHE. Correction to photoelectron reference energy ΔE and the obtained R-factor that characterizes fit quality are also reported. Uncertainty of the last digit is reported in parentheses.

**Sample**	**Cu foil**	**Cu_2O**	**CuO**	**$Au_{0.4}/Cu_2O$ NC**		**$Au_{0.8}/Cu_2O$ NC**		**$Au_{1.1}/Cu_2O$ NC**		**$Au_{2.7}/Cu_2O$ NC**	
				**as prepared**	**during CO_2RR**	**as prepared**	**during CO_2RR**	**as prepared**	**during CO_2RR**	**as prepared**	**during CO_2RR**
**N_{Cu-O}**	0	2		3.6(5)	-	3.3(2)	-	3.4(4)	-	2.96(17)	-
**R_{Cu-O} / Å**	-	1.847(3)		1.857(13)	-	1.877(6)	-	1.859(10)	-	1.858(6)	-
**σ^2_{Cu-O} / Å²**	-	0.0008(5)		-0.002(2)	-	0.002(1)	-	0.002(2)	-	0.003(1)	-
**N_{Cu-Cu}**	12	0		-	10(2)	-	11.9(9)	-	11(2)	-	9.8(7)
**R_{Cu-Cu} / Å**	2.545(4)	-		-	2.558(13)	-	2.539(5)	-	2.527(12)	-	2.539(5)
**σ^2_{Cu-Cu} / Å²**	0.0093(4)	-		-	0.007(2)	-	0.0096(7)	-	0.010(2)	-	0.009(1)
**ΔE / eV**	3.37	1.3(4)		2.0(16)	5(2)	4.3(8)	3.3(7)	2.3(13)	2(2)	0.6(7)	-2.8(8)
**R factor**	0.3 %	0.02 %		6.5%	4.4%	0.1%	0.5%	0.4%	3.6%	0.1%	0.6%

Table S11. Structural parameters (coordination number N, interatomic distances R, disorder factors σ^2) obtained from fitting the experimental Au L_3-edge EXAFS data acquired in the as prepared state and during CO_2RR at -1.0 V vs. RHE. Correction to photoelectron reference energy ΔE and the obtained R-factor that characterizes fit quality are also reported. Uncertainty of the last digit is reported in parentheses.

**Sample**	**Au foil**	**CuAu Alloy**	**$Au_{0.4}/Cu_2O$ NC**		**$Au_{0.8}/Cu_2O$ NC**		**$Au_{1.1}/Cu_2O$ NC**		**$Au_{2.7}/Cu_2O$ NC**	
		(from Ref.[10])	**as prepared**	**during CO_2RR**	**as prepared**	**during CO_2RR**	**as prepared**	**during CO_2RR**	**as prepared**	**during CO_2RR**
**N_{Au-Cu}**	0	6.8(6)	-	9(4)	-	8(2)	-	9(4)	-	10(6)
**R_{Au-Cu} / Å**	0	2.700(2)	-	2.610(6)	-	2.62(1)	-	2.61(2)	-	2.63(1)
**σ^2_{Au-Cu} / Å^2**	0	0.006(1)	-	0.011(3)	-	0.010(1)	-	0.011(4)	-	0.012(6)
**N_{Au-Au}**	12	8.6(9)	12(3)	3(4)	12(2)	4(2)	13(3)	3(4)	13(2)	2(6)
**R_{Au-Au} / Å**	2.860(4)	2.811(7)	2.83(2)	2.72(5)	2.84(9)	2.79(4)	2.83(1)	2.74(5)	2.84(1)	2.72(7)
**σ^2_{Au-Au}/ Å^2**	0.0084(8)	0.006(1)	0.009(3)	0.01(4)	0.009(2)	0.01(1)	0.011(2)	0.01(2)	0.009(2)	0.01(5)
**ΔE / eV**	2.1(5)	-1.6(4)	1.82(1.7)	-0.39(1.)	1.62(1.0)	2(2)	1.49(1.4)	-0.22(2)	1.59(1.2)	2(2)
**R factor**	0.25	0.062	2.65	0.19	0.989	0.089	2.13	0.25	1.39	0.51

Table S12. Register of the most important Au-Cu and Ag-Cu catalysts forming C_{2+} products under CO_2RR.

	Catalyst	Main C_{2+} products	authors explanation	Ref
		**CuAu catalysts**		
**Alloy**	**$AuCu_3$ alloy**	<1% FE C_{2+}, <2% FE ethanol	Synergistic effects	11
**Mixed systems**	**CuAu alloy NP, embedded in Cu**	30% FE ethanol, 16% C_2H_4	Electronic structure effects	12
**Au NP on Cu or Cu_2O**	**Au (30-50nm) on Cu_2O nanowires**	23% FE ethanol, 38.7% FE C_2H_4	Sequential catalysis	13
	**CuAu (3-5nm Au on polyCu)**	12% FE ethanol, 18.3% FE C_{2+}	Synergistic effects	14
	**Au/Cu_2O NC**	60% C_{2+}, 20% FE liquids	Sequential catalysis	This work
		**CuAg catalysts**		
**Mixed systems**	**CuAg**	41% FE ethanol		15
	**Cu95Ag5 thin film**	9% FE ethanol, 17% C_2H_4	Miscibility between Cu an Ag	16
	**Ag/Cu_2O_{PB}**	35% FE ethanol, 10% FE C_2H_4	Miscibility of Cu and Ag	17
**Ag NP on Cu_2O**	**Ag/Cu_2O**	18% FE ethanol, 52% FE C_2H_4	Spillover mechanism	18
	**5-Ag/Cu_2O NC**	17% FE ethanol, 34% FE C_2H_4	Partial Cu_2O reduction, Ag redispersion, Ag/Cu miscibility	7

REFERENCES

1. H. S. Jeon, J. Timoshenko, C. Rettenmaier, A. Herzog, A. Yoon, S. W. Chee, S. Oener, U. Hejral, F. T. Haase and B. Roldan Cuenya, *Journal of the American Chemical Society*, 2021, **143**, 7578-7587.
2. C. Zhan, F. Dattila, C. Rettenmaier, A. Bergmann, S. Kühl, R. García-Muelas, N. López and B. Roldan Cuenya, *ACS Catalysis*, 2021, **11**, 7694-7701.
3. X. W. Liu, F. Y. Wang, F. Zhen and J. R. Huang, *RSC Advances*, 2012, **2**, 7647-7651.
4. N. Leonard, W. Ju, I. Sinev, J. Steinberg, F. Luo, A. S. Varela, B. Roldan Cuenya and P. Strasser, *Journal*, 2018, **9**, 5064-5073.
5. B. Ravel and M. Newville, *Journal*, 2005, **12**, 537-541.
6. M. Newville, *Journal*, 2001, **8**, 322-324.
7. A. Herzog, A. Bergmann, H. S. Jeon, J. Timoshenko, S. Kühl, C. Rettenmaier, M. Lopez Luna, F. T. Haase and B. Roldan Cuenya, *Angewandte Chemie - International Edition*, 2021, **60**, 7426-7435.
8. Z. Xu, E. Lai, Y. Shao-Horn and K. Hamad-Schifferli, *Chemical Communications*, 2012, **48**, 5626-5628.
9. D. Friebel, F. Mbuga, S. Rajasekaran, D. J. Miller, H. Ogasawara, R. Alonso-Mori, D. Sokaras, D. Nordlund, T. C. Weng and A. Nilsson, *Journal of Physical Chemistry C*, 2014, **118**, 7954-7961.
10. A. I. Frenkel, V. S. Machavariani, A. Rubshtein, Y. Rosenberg, Y. Rosenberg, A. Voronel and E. A. Stern, *Physical Review B - Condensed Matter and Materials Physics*, 2000, **62**, 9364-9371.
11. D. Kim, J. Resasco, Y. Yu, A. M. Asiri and P. Yang, *Nature Communications*, 2014, **5**, 4948.
12. S. Shen, X. Peng, L. Song, Y. Qiu, C. Li, L. Zhuo, J. He, J. Ren, X. Liu and J. Luo, *Small*, 2019, **15**, 1902229.
13. J. Gao, D. Ren, X. Guo, S. M. Zakeeruddin and M. Grätzel, *Faraday Discussions*, 2019, **215**, 282-296.
14. C. G. Morales-Guio, E. R. Cave, S. A. Nitopi, J. T. Feaster, L. Wang, K. P. Kuhl, A. Jackson, N. C. Johnson, D. N. Abram, T. Hatsukade, C. Hahn and T. F. Jaramillo, *Nature Catalysis*, 2018, **1**.
15. Y. C. Li, Z. Wang, T. Yuan, D. H. Nam, M. Luo, J. Wicks, B. Chen, J. Li, F. Li, F. P. G. De Arquer, Y. Wang, C. T. Dinh, O. Voznyy, D. Sinton and E. H. Sargent, *Journal of the American Chemical Society*, 2019, **141**, 8584-8591.
16. D. Higgins, A. T. Landers, Y. Ji, S. Nitopi, C. G. Morales-Guio, L. Wang, K. Chan, C. Hahn and T. F. Jaramillo, *ACS Energy Letters*, 2018, **3**, 2947-2955.
17. S. Lee, G. Park and J. Lee, *ACS Catalysis*, 2017, **7**, 8594-8604.
18. J. Gao, H. Zhang, X. Guo, J. Luo, S. M. Zakeeruddin, D. Ren and M. Grätzel, *Journal of the American Chemical Society*, 2019, **141**, 18704-18714.
